# Optimized LC-MS method for simultaneous polyamine profiling and ADC/ODC activity quantification and evidence that ADCs are indispensable for flower development in tomato

**DOI:** 10.3389/fpls.2025.1636076

**Published:** 2025-09-11

**Authors:** Erin Samantha Ritchie, Edda von Roepenack-Lahaye, Dennis Perrett, Dousheng Wu, Thomas Lahaye

**Affiliations:** ^1^ ZMBP - General Genetics, University of Tübingen, Tuebingen, Germany; ^2^ ZMBP – Central Facilities, University of Tübingen, Tuebingen, Germany; ^3^ Hunan Key Laboratory of Plant Functional Genomics and Developmental Regulation, College of Biology, Hunan University, Changsha, China

**Keywords:** polyamines, arginine decarboxylase, ornithine decarboxylase, LC-MS, ADC, ODC, tomato, flower development

## Abstract

**Introduction:**

Polyamines (PAs) are essential for plant development and stress responses, requiring tight homeostatic regulation. As many PA enzymes are regulated post-transcriptionally, transcript-based methods cannot accurately predict protein abundance. This limitation highlights the need for alternative approaches to study PA homeostasis.

**Methods:**

We optimized a liquid chromatography–mass spectrometry (LC-MS)–based method to simultaneously quantify the activities of two key PA-synthesizing enzymes—arginine decarboxylase (ADC) and ornithine decarboxylase (ODC)—from plant tissues using stable isotope substrates. Substrate concentrations were optimized to increase assay sensitivity, and the method was adapted for *Nicotiana benthamiana* as a heterologous expression system.

**Results:**

In tomato leaf tissue, assay sensitivity improved more than tenfold. In *N. benthamiana*, expression of epitope-tagged ADCs revealed a direct correlation between protein abundance and enzymatic activity, indicating that ADC activity can infer native protein abundance. Proof-of-principle experiments confirmed substrate specificity of tomato ADC and ODC enzymes and identified essential catalytic residues of tomato ADCs. The protocol was further expanded to quantify 11 PA-network metabolites from the same LC-MS sample—six more than previously reported—providing a comprehensive overview of PA metabolism when visualized as a pathway heatmap. LC-MS analysis of tomato CRISPR–Cas9 mutants deficient in ADC or ODC, revealed that the *adc1/adc2* double mutant had no detectable agmatine and reduced putrescine, whilst spermidine and spermine remained unaffected. Phenotypic analysis showed severe developmental defects in this mutant, including complete flower loss, underscoring the indispensable role of ADCs in flower development.

**Discussion:**

Together, the optimized LC-MS method, the ability to functionally analyze recombinant ADC/ODC proteins *in planta*, and the use of tomato CRISPR mutants provide a versatile toolkit to dissect PA homeostasis and PA-dependent developmental processes in plants.

## Introduction

Polyamines (PAs) are essential metabolites for almost all organisms (Michael, 2016). In plants they are required for various processes such as growth, development, reproduction, senescence, and play key roles in abiotic and biotic stress responses (Alcázar et al., 2010; Chen et al., 2019; Gonzalez et al., 2021; Napieraj et al., 2023; Blázquez, 2024). PAs are low molecular weight compounds consisting of at least two amino groups and exist in various forms: free, bound to cellular moieties (e.g., RNA, DNA, and cell wall components), or conjugated to other metabolites such as hydroxycinnamic acids (Bassard et al., 2010; Chen et al., 2019; Pál et al., 2021). Despite their indispensability and numerous roles, the mechanism by which PA homeostasis is maintained remain unclear.

**Figure 1:**
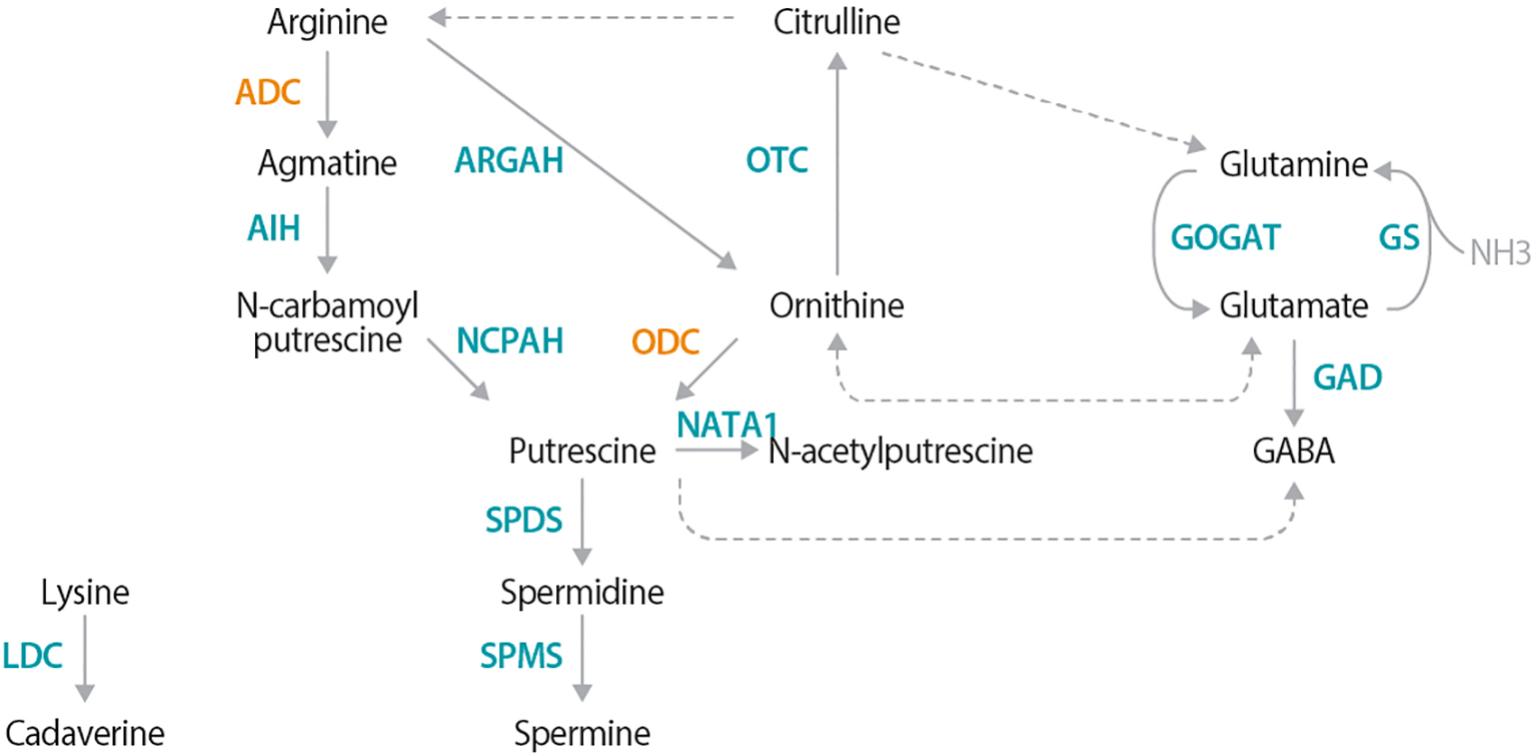
Polyamine metabolic pathway, highlighting the ADC- and ODC dependent pathways. Synthesis of the main plant polyamines (PAs), putrescine, spermidine, and spermine originates with either arginine via the arginine decarboxylase (ADC)-dependent route or the ornithine route via ornithine decarboxylase (ODC). Cadaverine, another PA, is produced from lysine via lysine decarboxylase (LDC). Solid arrows represent reactions catalyzed by a single enzyme, while dotted arrows indicate multiple enzymatic steps. Additional enzyme names are as follows: agmatine iminohydrolase (AIH); N-carbamoylputrescine amidohydrolase (NCPAH); spermidine synthase (SPDS); spermine synthase (SPMS); arginase (ARGAH); ornithine-carbamoyl transferase (OTC); glutamate synthase (GOGAT); glutamine synthase (GS); glutamate decarboxylase (GAD); and N-acetyltransferase activity 1 (NATA1).

In plants, PAs are synthesized via two main routes; both converging on the production of putrescine (**Figure 1**; Liu et al., 2015; Michael, 2016). The first anabolic route involves decarboxylation of arginine into agmatine by arginine decarboxylase (ADC), while the second involves direct decarboxylation of ornithine into putrescine by ornithine decarboxylase (ODC). Putrescine is then converted to the higher PAs, spermidine and spermine. Cadaverine, another PA, is independently synthesized from lysine-by-lysine decarboxylase (LDC) (Bunsupa et al., 2012; Jancewicz et al., 2016). ADC, ODC, and LDC enzymes belong to the same IV pyridoxyl 5-phosphate (P5P)-dependent decarboxylase family (Sandmeier et al., 1994). These enzymes dimerize head-to-tail configuration, with essential lysine and cysteine residues from each monomer forming two catalytic sites at the interface; the lysine interacting with P5P, and the cysteine responsible for substrate specificity (Poulin et al., 1992; Hanfrey et al., 2001; Bunsupa et al., 2012; Liang et al., 2019).

Although *Arabidopsis thaliana* is a commonly used plant model for genetic and biochemical studies, it is not suitable for investigating canonical PA metabolism which requires both ADC and ODC. This is because many *Brassicaceae* species, including *A. thaliana* lack the PA anabolic enzyme ODC, and therefore rely solely on the ADC-dependent pathway for PA synthesis (Hanfrey et al., 2001; Jiménez-Bremont et al., 2014). As a result, an *A. thaliana adc1/adc2* double mutant is embryonically lethal (Urano et al., 2005), thereby preventing studies in this model plant that could provide insights into which biochemical or developmental processes depend on the functionality of ADC1/2 proteins.

In contrast to *A. thaliana* and some other Brassicaceae species, solanaceous species (e.g., tomato [*Solanum lycopersicum*; *Sl*] and *Nicotiana benthamiana*) possess the canonical PA biosynthetic pathway found in most land plants. Additionally, *N. benthamiana* enables rapid functional studies of proteins and their mutant derivatives *in planta* as it can be efficiently transiently transformed using *Agrobacterium tumefaciens* (Ranawaka et al., 2023; Golubova et al., 2024). Moreover, *N. benthamiana* can serve as a host for a wide range of model plant pathogens (Goodin et al., 2008; Schultink et al., 2017; Qi et al., 2018), making it an ideal platform to study the mechanistic principles of PA metabolism and its relevance in plant-pathogen interactions.

For decades, plant PAs have been measured using chromatography-based methods (Richards and Coleman, 1952; Smith and Richards, 1962; Smith, 1970), with recent analytical advancements enabling more accurate PA separation using high-performance liquid or gas chromatography. More recently, methods based on liquid chromatography-mass spectrometry (LC-MS) have been developed for quantification of plant PAs without derivatization, significantly reducing time and cost, and thereby greatly improving the suitability of these approaches for high-throughput analysis (Häkkinen et al., 2007; Sánchez-López et al., 2009). However, most studies solely quantified putrescine, spermidine, and spermine, neglecting pathway precursors and breakdown products. The lack of comprehensive quantitative information on the concentrations of key metabolites involved in anabolic and catabolic PA metabolism represents a major limitation that has hindered a broader understanding of the regulatory principles underlying PA homeostasis.

In addition to studies quantifying PA metabolites, there have also been investigations into the enzymatic activities of enzymes involved in PA homeostasis, with particular focus on the key enzymes ADC and ODC (Smith and Richards, 1962; Smith, 1970; Kaur-Sawhney et al., 1982; Rossi et al., 2018). In the past, the enzymatic activity of ADC and ODC was often determined by supplying radioactive substrates and measuring the release of radioactive CO_2_, rather than quantifying the enzyme-specific metabolic products. However, approaches quantifying ADC/ODC activity based on CO_2_ release are limited, as arginine and ornithine pools are metabolically interconnected (Winter et al., 2015; Majumdar et al., 2016; Joshi and Fernie, 2017). As a result, one labeled substrate can be rapidly converted into another, meaning detection of released radioactive CO_2_ might not be due to the activity of a single enzyme but rather the combined activities of ADC, ODC, and potentially other enzymes involved in PA homeostasis. These considerations highlight that elucidating the regulatory networks underlying PA homeostasis requires analytical methods capable of simultaneously quantifying multiple enzyme activities and PA-related metabolites, methods which are not yet fully established.

**Figure 2:**
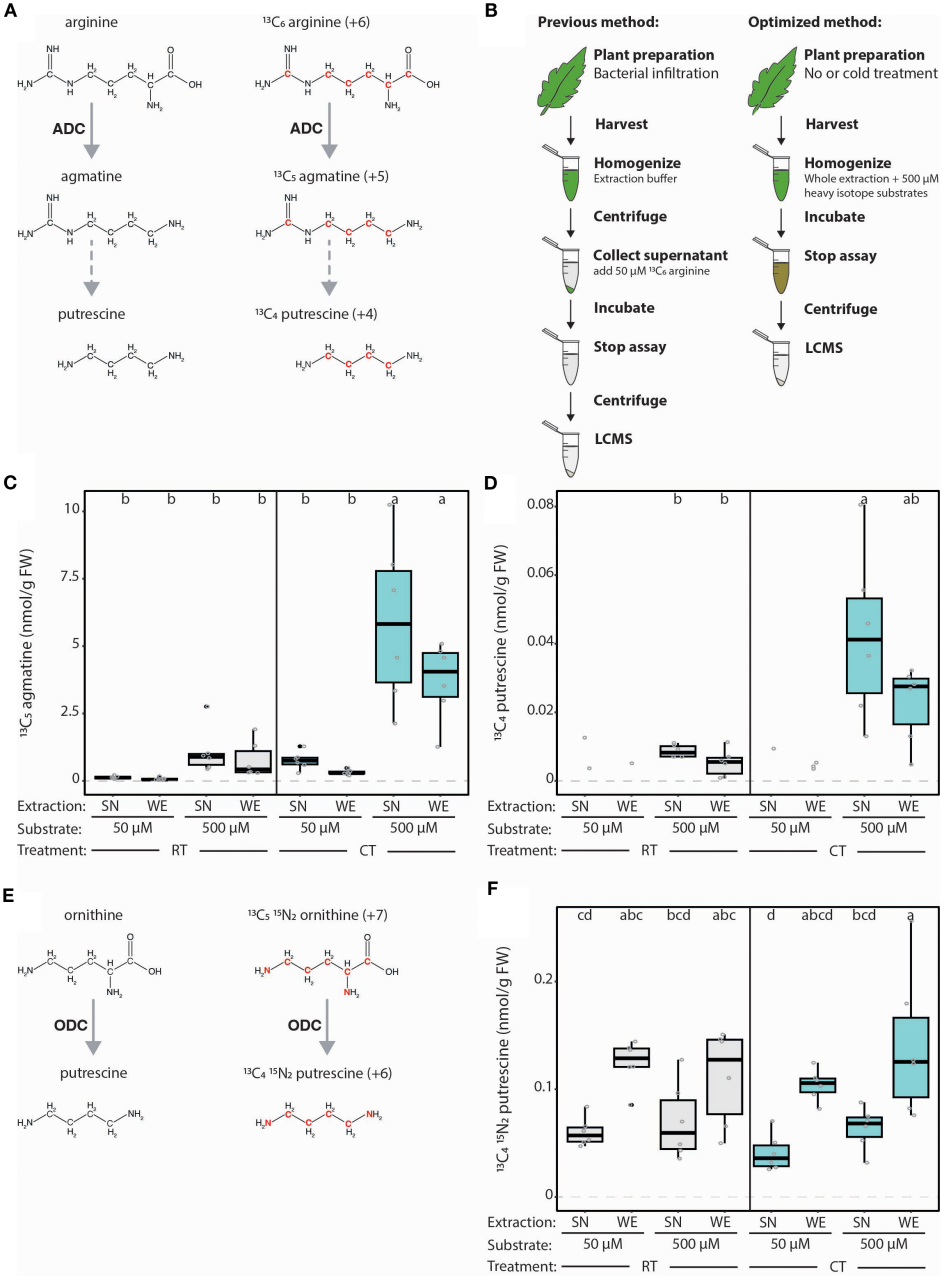
Optimization of LC-MS-based method for measuring ADC activity from plant extracts. **(A)** Schematic representation of the ADC-catalyzed conversion of natural (left) and heavy (right) isotope arginine variants into agmatine, and the subsequently generated putrescine variant. Red font indicates heavy atoms in these isotopes. **(B)** Illustration displaying previously established LC-MS-based quantification of ADC activity according to Wu et al., 2019 (left) and the optimized approach described here (right). In the previously established method, samples of tomato leaves were harvested, flash frozen, ground, homogenized in an extraction buffer, then centrifuged and the supernatant (SN) collected. 50 µM ^13^C_6_ arginine is added to each SN sample and incubated for 1 hour at 37°C before the assay is stopped by adding 5 µL of heptafluorobutyric acid (HFBA) and centrifuged (30 minutes, 4°C). In the optimized protocol, no initial centrifugation step is required; instead, heavy isotope substrates (500 µM) are added directly to the buffer used to homogenize the whole extracts (WE). **(C)** ADC activity is increased upon higher substrate concentration but not impacted by extraction procedure. ADC activity (^13^C_5_ agmatine in nmol/g of fresh weight [FW] tissue) in tomato leaf samples, pretreated either with room temperature (RT; grey) or cold treatment (CT; blue), with either 50 or 500 µM ^13^C_6_ arginine added to the SN or WE. **(D)** The synthesis of heavy putrescine is only detectable at high substrate concentrations. ^13^C_4_ putrescine (nmol/g FW), generated from ADC-produced ^13^C_5_ agmatine in the same samples presented in **(C)**. **(E)** Graphical representation of the ODC-catalyzed conversion of natural (left) and heavy (right) isotope ornithine variants into putrescine. Red font indicates heavy atoms in these isotopes. **(F)** ODC activity can successfully be simultaneously measured using LC-MS method. ODC activity (^13^C_4_^15^N_2_ putrescine in nmol/g FW) in the same samples presented in **(C, D)**. For **(C, D, F)**, all concentrations were normalized to the internal D_5_ tryptophan control. Statistical significance was determined using ANOVA followed by Tukey’s *post hoc* HSD test; different letters above each box indicate statistically significantly groups (α = 0.05). Each biological replicate is represented by a grey circle; n = 6. If a metabolite could not be detected in all samples, only the remaining replicates are displayed without a boxplot and this group was removed from the statistical analysis.

Recently, we developed an LC-MS approach to simultaneously quantify ADC activity and the levels of five PA-network metabolites from the same plant sample (Wu et al., 2019; Gallas et al., 2024). To monitor ADC activity specifically, a stable (non-radioactive) heavy isotope variant of arginine (^13^C_6_ arginine) was added to plant extracts, and the formation of the derived heavy isotopic product, ^13^C_5_ agmatine was quantified as a readout of ADC enzymatic activity (**Figure 2A**). Here, we present our optimized LC-MS-based method, in which the optimization of heavy isotope substrate concentrations, extraction protocols, LC-MS gradient, and metabolite analysis has led to improved sensitivity, a simplified and less laborious workflow, and the ability to detect 11 PAs in a single MS run. We further optimized our assay to enable the simultaneous quantification of both ADC and ODC enzyme activities by using different isotope-labeled metabolites. These optimization experiments were also conducted on tomato leaves following a cold treatment (CT) – a condition that transcriptionally induces tomato *ADC*s (Upadhyay et al., 2020) – to not only present how PA metabolism is influenced following an abiotic stress, but to also highlight the usability of this method.

Additionally, we showcase our LC-MS method by conducting ADC activity and PA profiling on four CRISPR-Cas9-generated tomato mutants, including an *adc1/adc2* double mutant, and characterize their phenotypes. Through this, we demonstrate the importance of ADCs for PA metabolism and their crucial role in plant and flower development.

## Materials and methods

### Plant materials and growth conditions

Tomato (Moneymaker) and *N. benthamiana* were grown at 21 ± 3°C, 30-50% humidity, and a 16/8-hour light/dark photoperiod.

### CRISPR Cas9 mutagenesis of tomato *ADC* and *ODC* genes

Previously created tomato single *adc1* and *adc2* and double *adc1/adc2* mutants (Wu et al., 2019) and the new *odc1* mutant were generated via CRISPR-Cas9 (Jacobs et al., 2015) using two gRNAs targeting each gene. These constructs were transformed into tomato (Wittmann et al., 2016) and genotyping was conducted as described by Wu et al., 2019 using primers presented in **Supplementary Table 1**.

### Phenotyping tomatoes

Germinated seedlings were genotyped, then transferred into 11 cm diameter pots at 18 days after germination (DAG), and phenotypes measured/counted every 3 days from 26-47 DAG. Plant height was measured to nearest 0.5cm from the cotyledon to shoot apical meristem. Only fully expanded adult leaves originated from the primary stem were counted. All buds and opened flowers were counted from each plant. Photos of every replicate were taken 37 DAG and leaf and flower photos taken at 47 DAG.

### Cold treatment of tomato leaves

The youngest, fully expanded adult leaf was cut from each replicate (aged 4-5 weeks). The terminal and two primary leaflets were kept in water at ~6°C (cold treatment; CT) or room temperature (RT) for 24 hours (in darkness). Samples for LC-MS or RNA extraction were taken without allowing CT leaves to warm.

### Generation of expression constructs

Tomato *ADC1*, *ADC2*, and *ODC1* genes were cloned from WT tomato gDNA into pUC57 vectors as level I modules for further Golden Gate cloning (Binder et al., 2014). PCR-based site-directed mutagenesis of key SlADC catalytic residues was conducted in level I modules, replacing nucleotides to encode an arginine instead of the WT lysine or cystine. Constructs were cloned into level II T-DNA expression vectors (Binder et al., 2014) and transformed into *A. tumefaciens GV3101* using electroporation.

### Transient expression in *N. benthamiana*

*A. tumefaciens* strains containing T-DNA expression constructs and a p19 silencing suppressor plasmid-containing strain were grown in Yeast Extract Broth (YEB) media (28°C, 180rpm), centrifuged, and resuspended in infiltration buffer (10 mM MgCl_2_, 5 mM MES, and 150µM acetosyringone) to an optical density at 600nm (OD_600_) of ~0.5. After an additional incubation (~4 hours, 28°C, 180rpm), all expression construct-containing cultures were mixed 1:1 with the p19-containing strain to a final OD_600_ of 0.4 and infiltrated into the abaxial surface of *N. benthamiana* leaves using a blunt end syringe.

### ADC and ODC activity assay sample preparation

50mg (exact weight recorded) of plant tissue was flash frozen, ground in pre-cooled adapters using a TissueLyser II (Qiagen ^®^; 22 Hz, 30 sec, x2) and homogenized in ADC activity buffer (300µL of 5 mM Tris [pH 7.5], 0.75% polyvinylpolypyrrolidone [PVPP], 1 mM ascorbate, 50 µM pyridoxal 5 phosphate [P5P], 0.5 x protease inhibitor [Roche complete]; 2µM ^13^C_1_ D_2_ citrulline [Eurisotop^®^] and 2.5µM D_5_ tryptophan [Sigma-Aldrich^®^] as internal standards). Samples were then centrifuged (10min, 4°C, 18000g) and the supernatant (SN) collected. 10 µL of each ADC and ODC enzyme substrates ^13^C_6_ arginine [Carl Roth^®^] and ^13^C_5_^15^N_2_ ornithine [Eurisotop^®^] were then added to the SN of each sample (180 µl) to a final concentration of 50 or 500µM and incubated for 1 hour at 37°C before the assay was stopped by using 5µL of heptafluorobutyric acid (HFBA) and centrifuged (30 min, 4°C, 18000g). For the “whole extract” (WE) protocol, the initial centrifugation step and collection of SNs was omitted, with the heavy isotope enzyme substrates directly included in the ADC activity buffer. When testing for LDC activity, 500 µM ^13^C_6_^15^N_2_ lysine (LGC Standards^®^) was included in the buffer.

For *N. benthamiana* samples, which require an additional extraction step, 80µL of the stopped WE assay samples was combined with 200µL methanol and 100µL chloroform, and incubated (10 minutes, 25°C, 950 rpm). 120µL of ultrapure MilliQ water was added, the incubation repeated, a final centrifugation step (18,000 g, 30 minutes, 4°C), and the upper phase used for profiling.

### LC-MS profiling

To determine if metabolite levels fell into the linear range of their respective calibration curves (**Supplementary Table 2**), dilutions (in water, 0.1% FA, 0.1% HFBA) of representative samples were tested prior to running all samples. LC-MS profiling was performed using a Micro-LC M5 (Trap and Elute) and QTRAP6500+ (Sciex) system operated in Multiple Reaction Monitoring (MRM) mode. Chromatographic separation was achieved on a HaloFused C18 micro column (150 x 0.5 mm; 2.7 μm; AB Sciex) and a Luna C18 (2) micro trap column (5μm; 100 Å; 20×0.5mm; Phenomenex); injection volume was 50 μL. Analytes were ionized using an Optiflow Turbo V ion source equipped with a SteadySpray T micro electrode (10-50 μL min^-1^) in positive ion mode. **Supplementary Table 3** presents information concerning the chromatographic gradients and MS parameters. A shorter trap gradient was chosen for the assays without HFBA in the solvent to reduce the loss of the ion paring agent in the sample itself. A general loss of sample volume of about 10% (data not shown) using the reduced trapping time was deemed acceptable.

Vendors Sciex OS software was used to analyze acquired MRM data. The metabolite content in each replicate was calculated using commercial standards (external calibration in water, 0.1% FA, 0.1% HFBA), except for ^13^C_5_ agmatine, ^13^C_4_ putrescine, and ^13^C_4_^15^N_2_ putrescine, which were quantified based on their natural variants. Data were normalized against exact sample fresh weight (FW), and D_5_ tryptophane or ^13^C_1_D_2_ citrulline.

*Note:* A small ^13^C_5_ agmatine peak was observed in control assays with no plant extracts. As no enzyme was present in these samples, the production of ^13^C_5_ agmatine can only be explained by chemical decomposition or insource fragmentation of ^13^C_6_ arginine within the MS ion source. This is further confirmed by the retention time of this ^13^C_5_ agmatine peak, which is not congruent to the natural agmatine but rather to arginine. All samples with low ADC activity (e.g., tomato *adc1/adc2* double mutant extracts) were double-checked for accumulation of enzymatically produced ^13^C_5_ agmatine (retention time of agmatine) instead of the chemically derived variant (retention time of arginine) (**Supplementary Figure 1**).

### RT-qPCR

RNA was extracted from flash frozen tomato samples using the Universal RNA Purification Kit (Roboklon GmbH); on- and off-column DNase (ThermoFischer Scientific) treatments were both conducted as tomato *ADC*s do not contain introns. RNA (850 ng) was converted to cDNA using the RevertAid cDNA Synthesis Kit (ThermoFischer Scientific) and diluted 1:10 in RNA-free water. RT-qPCRs were conducted as described by Wu et al., 2019, using the CFX96 system (BioRad), and 3 technical replicates per biological replicate. *SlADC1* and *SlADC2* transcript levels were calculated using 2^-ΔΔCT^ and normalized to *SlTIP41* (Lacerda et al., 2015) expression as previously described by Wu et al., 2019. RT-qPCR primers used in this work are found in **Supplementary Table 1**.

### Immunodetection of epitope-tagged proteins

*N. benthamiana* leaf tissue was flash frozen and ground in pre-cooled adapters, then homogenized in extraction buffer (80 mM Tris [pH 7.5], 2.5% SDS, 50 mM EDTA, 2.5x strength protease inhibitor; 33 µL per leaf disk) and loading dye added (20 mM Tris-HCl pH 8.6, 40% [v/v] glycerol, 10% [v/v] 2-mercaptoethanol, and 0.1% [w/v] bromophenol blue; 13 µL per leaf disk). Samples were heated (90°C, 10 minutes), briefly centrifuged, and loaded onto a 10% SDS PAGE gel. Proteins were transferred to a PVDF membrane using Trans-Blot^®^ Turbo Transfer System (BioRad), blocked (5% milk powder in TBST [50 mM Tris-HCl, 150 mM NaCl, 0.05% Tween-20]), then incubated overnight (6°C, rocking) with the epitope-detecting antibody (i.e., monoclonal, conjugated anti-HA-HRP antibody [Roche] or monoclonal anti-c myc [raised in mouse; Sigma Aldrich]). Washed blots (3x 5 min in TBST) were either imaged directly or blocked, incubated with a conjugated anti-mouse-HRP antibody (Sigma Aldrich) for 2 hours, and washed again. ECL Select™ Western Blotting Detection Reagent (Amersham™, cytiva) was used for detection and blots were imaged using an Amersham™ Imager 600, GE Healthcare Life Sciences. Ponceau staining (0.1% [w/v] Ponceau S, and 5% [v/v] glacial acetic acid) of all blots was conducted to verify even protein loading.

### Data analysis and visualization

Microsoft Excel and R were used for basic statistical analysis and generation of graphs and heatmap diagrams, which were then assembled using Adobe Illustrator. Code to generate metabolite heatmaps is available: https://github.com/TheDepe/Metabolite-Measurement.

## Results

### Optimizating the LC-MS-based method for ADC activity quantification

We first sought to improve the sensitivity of our LC-MS-based method to quantify ADC activity and PA-related metabolites from plant extracts, even with low *ADC* expression. In the previously established protocol, ADC activity was quantified in the supernatant (SN) of cellular extracts (**Figure 2B**; Wu et al., 2019). We rationalized that SNs may lack organelles containing ADC proteins and metabolites (e.g., chloroplasts), consequently reducing ADC activity. Therefore, we compared ADC activity in SNs (i.e., previous protocol) to whole cellular extracts (termed here on out as ‘whole extract’ or WE) where the extraction buffer is applied to ground leaf tissue without an additional centrifugation step (**Figure 2B**). Furthermore, we considered whether a higher ADC substrate (i.e., ^13^C_6_ arginine; **Figure 2A**) concentration would elevate ADC activity. We therefore compared using ten times more ^13^C_6_ arginine (i.e., 500 µM; “high”) to the original 50 µM (“low”) concentration (**Figure 2B**).

High versus low concentrations of the ADC substrate were added to the SN or WE of tomato leaves kept at either room temperature (RT) or cold-treated (CT); the latter is known to elevate *SlADC* transcript levels. Indeed, both *SlADC1* and *SlADC2* transcript levels were significantly induced by ~3.5- and 2.9-fold following CT relative to RT controls (**Supplementary Figure 2**). When using the higher ^13^C_6_ arginine concentration, we observed a dramatic increase in ADC activity (**Figure 2C**). While this was particularly evident in CT samples with induced *SlADC* levels (~11-fold for WE and ~7-fold for SN samples), a similar increase was observed in RT samples (i.e., ~11- and ~8-fold higher in WE and SN samples, respectively), although not statistically significant (**Figure 2C**). Interestingly, applying a higher ^13^C_6_ arginine concentration enabled reliable detection of the heavy isotopic variant of putrescine produced from ^13^C_6_ arginine (i.e., ^13^C_4_ putrescine; **Figure 2A**), which was not detectable with the low ^13^C_6_ arginine concentration (**Figure 2D**). Overall, these results demonstrate that increasing the ^13^C_6_ arginine substrate concentration elevated the sensitivity of our ADC activity assay, enabling reliable measurement of ^13^C_5_ agmatine, and derived ^13^C_4_ putrescine, even under *ADC* non-inducing conditions.

Unexpectedly, we observed no significant differences in levels of ^13^C_5_ agmatine or ^13^C_4_ putrescine between SN and WE samples (**Figures 2C, D**). However, the SN-based ADC activity protocol is more labor-intensive, time-consuming, and error-prone due to more handling steps, making the new WE protocol preferable.

### Addition of ODC activity quantification to our LC-MS method

Measuring multiple enzyme activities in parallel reduces experimental costs and workload. Therefore, we wanted to test if both ADC and ODC activities (i.e., decarboxylation of ornithine to putrescine) could be quantified in one LC-MS run, despite both pathways producing putrescine (**Figure 1**). To achieve this, heavy isotopically labelled substrates of ADC and ODC were selected that would produce putrescine variants with sufficiently different molecular masses to be distinguishable by MS. For this, ^13^C_6_ arginine (+6 mass) and ^13^C_5_^15^N_2_ ornithine (+7 mass) were selected as ADC and ODC substrates, which produce ^13^C_4_ putrescine (+4) and ^13^C_4_^15^N_2_ putrescine (+6), respectively (**Figures 2A, E**).

We successfully detected ^13^C_4_^15^N_2_ putrescine in our tomato extracts and discriminated it from the ADC-derived ^13^C_4_ putrescine, enabling simultaneous ADC and ODC activity quantification (**Figure 2F**). In contrast to ADC activity, ODC activity was not elevated by higher substrate levels and ODC activity was higher in all WE assays compared to the SN. Our results indicate that a substantial portion of ODC enzymes are localized in organelles and suggest that WEs are preferable to SNs for assaying enzyme activities.

### The ion pairing agent HFBA, is not an essential component of the LC-MS gradient composition

We also considered the LC-MS gradient composition for optimization. Previously, 0.05% heptafluorobutyric acid (HFBA) was included in the gradient solvents as an ion pairing agent (Wu et al., 2019). However, due to its acidity, HFBA could cause corrosion of internal LC-MS components and also increase LC-MS contamination levels by adhering to the column and MS ion source. The assay from **Figure 2** was analyzed twice; once with HFBA in the solvent, and once without, relying solely on the HFBA added to the sample (i.e., to stop enzyme activity assay; **Figure 2B**) for ion pairing. In all cases, ^13^C_5_ agmatine intensities showed no significant difference between the two gradient compositions (**Supplementary Figure 3**). Therefore, HFBA can be omitted from the solvent without affecting activity quantification, whilst improving longevity and performance of the LC-MS system.

### Expanding the profile of quantifiable PA-related metabolites enables interpretation of metabolic fluxes after cold treatment

Monitoring changes across the wider PA network – particularly of anabolic compounds – would improve our understanding of PA metabolic fluxes during stress responses. Previously, we had only quantified the concentrations of agmatine, putrescine, spermidine, and spermine (Wu et al., 2019). Yet, to better comprehend the impact of abiotic stresses or extraction methods (e.g., SN and WE), we extended our protocol to include arginine, ornithine, citrulline, glutamine, N-acetylputrescine, cadaverine, and later, lysine. This was achieved by adjusting LC gradients to avoid co-elution of structural isomers such as acetyl-putrescine and agmatine, and glutamine and lysine (**Supplementary Tables 2**, **3**, **Supplementary Figure 4**).

**Figure 3:**
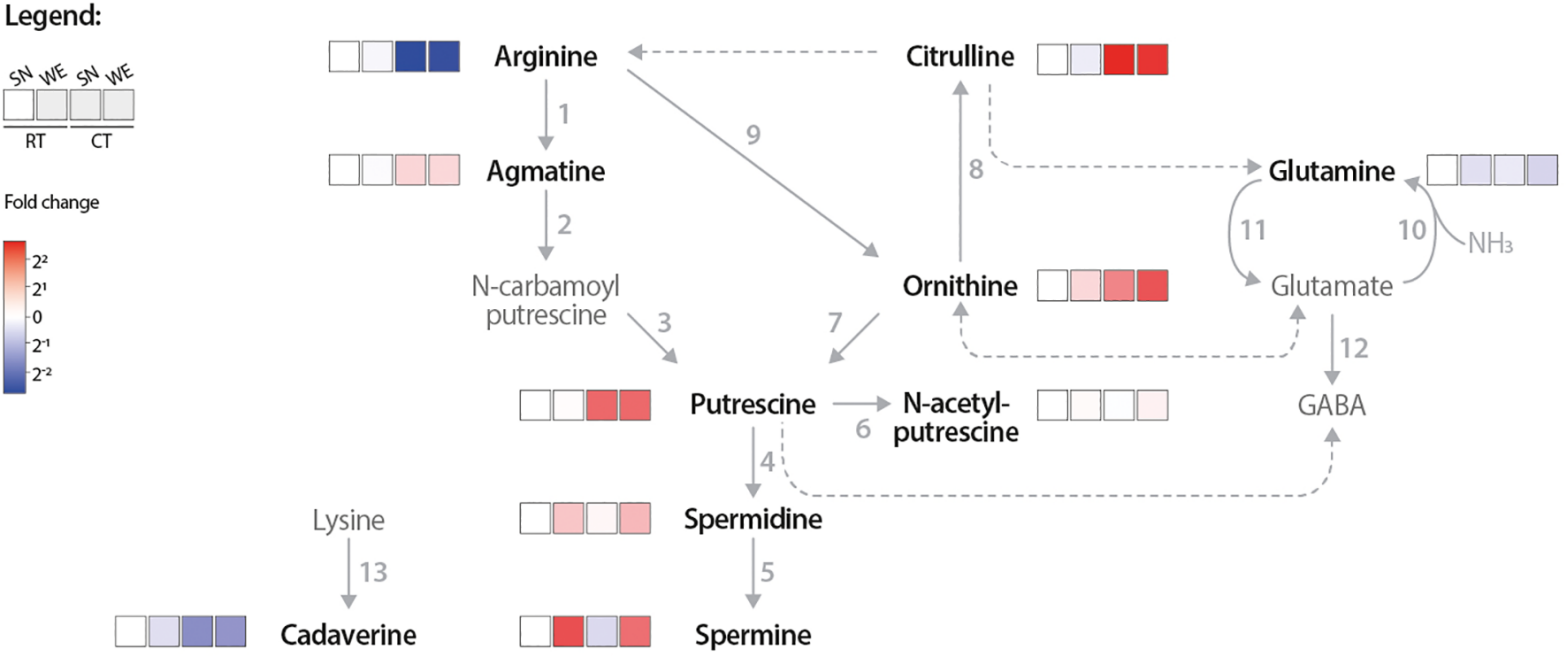
Pathway heatmap highlights how cold treatment influences PA network flux. PA and related metabolite concentrations were measured via LC MS from room temperature (RT) and cold treated (CT) tomato leaf samples from either supernatant (SN) or whole extraction (WE) assay samples. Only samples with 500 µM ^13^C_6_ arginine used are shown. The log2 fold change (FC) of each metabolite was calculated relative to the RT, SN-extracted (RT SN) condition; red indicates higher and blue lower metabolite levels compared to the RT SN. Solid arrows represent reactions catalyzed by a single enzyme, while dotted arrows indicate multiple enzymatic steps. Numbers correlate to the following enzymes: 1, ADC; 2, AIH; 3, NCPAH; 4, SPDS; 5, SPMS; 6, NATA1; 7, ODC; 8, OTC; 9, ARGAH; 10 GOGAT; 11, GS; 12, GAD; and 13, LDC. Only bold, black named metabolites were measured in this LC-MS experiment.

When quantifying changes in the concentrations of numerous metabolites in response to external (e.g. biotic or abiotic stress) or internal (e.g. developmental program) stimuli, or due to differences in extraction protocols (e.g., SN versus WE), it is difficult to infer metabolic fluxes if the data are displayed using conventional bar charts, as such representations do not reveal the causal relationships between metabolites. Therefore, we presented our LC-MS profiling results as a heatmap-based pathway diagram to better visualize metabolic relationships of the PA network (**Figure 3**). Following CT, agmatine, putrescine, ornithine, and citrulline levels increased, while arginine levels were reduced compared to the RT samples. This suggests that cold-induced *SlADC* transcript levels and ADC activity reduces the arginine pool to produce more agmatine and subsequently putrescine. Interestingly, spermidine and spermine levels are not influenced by CT, suggesting a regulatory mechanism that prevents the cold-induced increase in putrescine from causing an unregulated increase in these higher PAs.

Our results collectively show that a higher substrate concentration (i.e., 500 µM) increases assay sensitivity. Additionally, using WEs instead of SNs, now captures the activity of both cytoplasmic- and organelle-localized enzymes. We also found that omitting the ion pairing agent HFBA from the LC-MS gradient had no impact metabolite quantification but protects the LC-MS mechanical components. Finally, visualizing PA-related metabolite changes via a heatmap pathway diagram aids in understanding the interdependencies between metabolites.

### ADC protein abundance correlates with ADC activity

**Figure 4:**
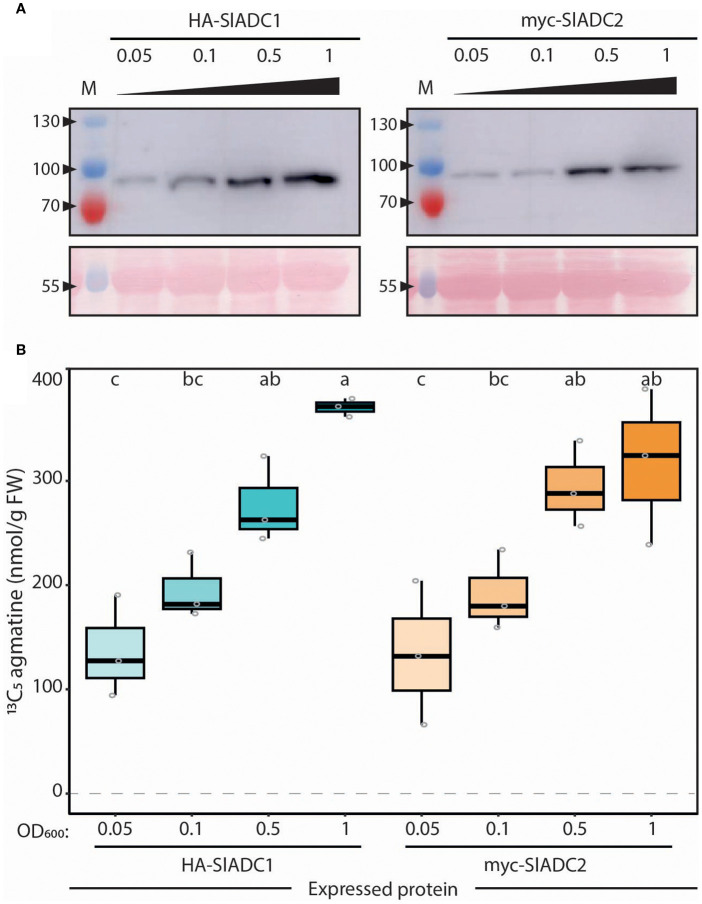
ADC activity directly and linearly correlates with ADC protein abundance. **(A)** ADC1 and ADC2 protein abundance increases with increasing bacterial load. Immunoblot analysis of *N. benthamiana* leaves agroinfiltrated with 35Sp: HA-SlADC1:35St (left) and 35Sp:myc-SlADC2:35St (right) at increasing OD_600_ (0.05, 0.1, 0.5, and 1) of samples taken 2 days post infiltration (dpi). Upper images (cropped) show HA-SlADC1 detection (left) and myc-SlADC2 (right), expected sizes are 78 and 80 kDa, respectively. Ponceau stained blots show equal protein loading (bottom). **(B)** ADC activity directly correlates with increasing ADC protein abundance. ADC activity (^13^C_5_ agmatine in nmol/g FW, normalized to D_5_ tryptophan) from the same *N. benthamiana* HA-SlADC1 (blue) and myc-SlADC2 (orange)-agroinfiltrated samples shown in A), with increasing color intensity representing increasing bacterial loads. Statistical significance was determined using an ANOVA followed by Tukey’s *post hoc* HSD test; different letters above each box indicate statistically significantly different groups (α = 0.05). Each biological replicate represented by a grey circle; n = 3.

Previously, we assumed a linear relationship between ADC protein abundance and its enzymatic activity; yet this assumption had not been experimentally validated (Wu et al., 2019; Gallas et al., 2024). To test for such a linear relationship, we expressed tomato ADC (SlADC) proteins in *N. benthamiana* leaves, where their expression levels can be easily modulated by inoculating varying amounts of T-DNA-containing *A. tumefaciens* strains. These T-DNA constructs encoded epitope-tagged SlADC1/2, driven by the constitutively active *35S* promoter. They were transformed into *A. tumefaciens* and infiltrated into *N. benthamiana* leaves (termed agroinfiltration) with increasing bacterial loads (i.e., OD_600_ 0.05, 0.1, 0.5, and 1). Two days post infiltration (dpi), samples were harvested for both immunoblotting and LC-MS profiling. Immunoblots showed increasing signal intensity of tagged SlADCs that mirrored the increasing bacterial loads (**Figure 4**).

Previously, unprocessed *N. benthamiana* leaf samples rapidly clogged the micro-LC columns. Therefore, an additional liquid-liquid extraction step was applied to all *N. benthamiana* leaf samples following the assay. Here, a proportion of alkaloids would be separated away from the upper phase that is subsequently used for LC-MS profiling, reducing their interference with the micro-LC columns.

Comparable to the protein immunodetection analysis, rising bacterial load increased ADC activity (**Figure 4**), confirming our hypothesis of a linear relationship between ADC abundance and activity. To confirm that this observation was not due to heightened biotic stress influencing ADC activity, the same experiment was conducted with epitope-tagged fluorophore controls. Again, increasing bacterial loads were associated with higher detectable epitope-tagged protein levels, but no mirrored increase in ADC activity was observed (**Supplementary Figure 5**). Overall, these results confirm a linear relationship between SlADC1/2 protein abundance and ADC activity, demonstrating that ADC activity quantification can be used as a reliable proxy for ADC protein levels from plant extracts.

### ADC activity quantification to test functionality of WT and mutant ADC variants

**Figure 5:**
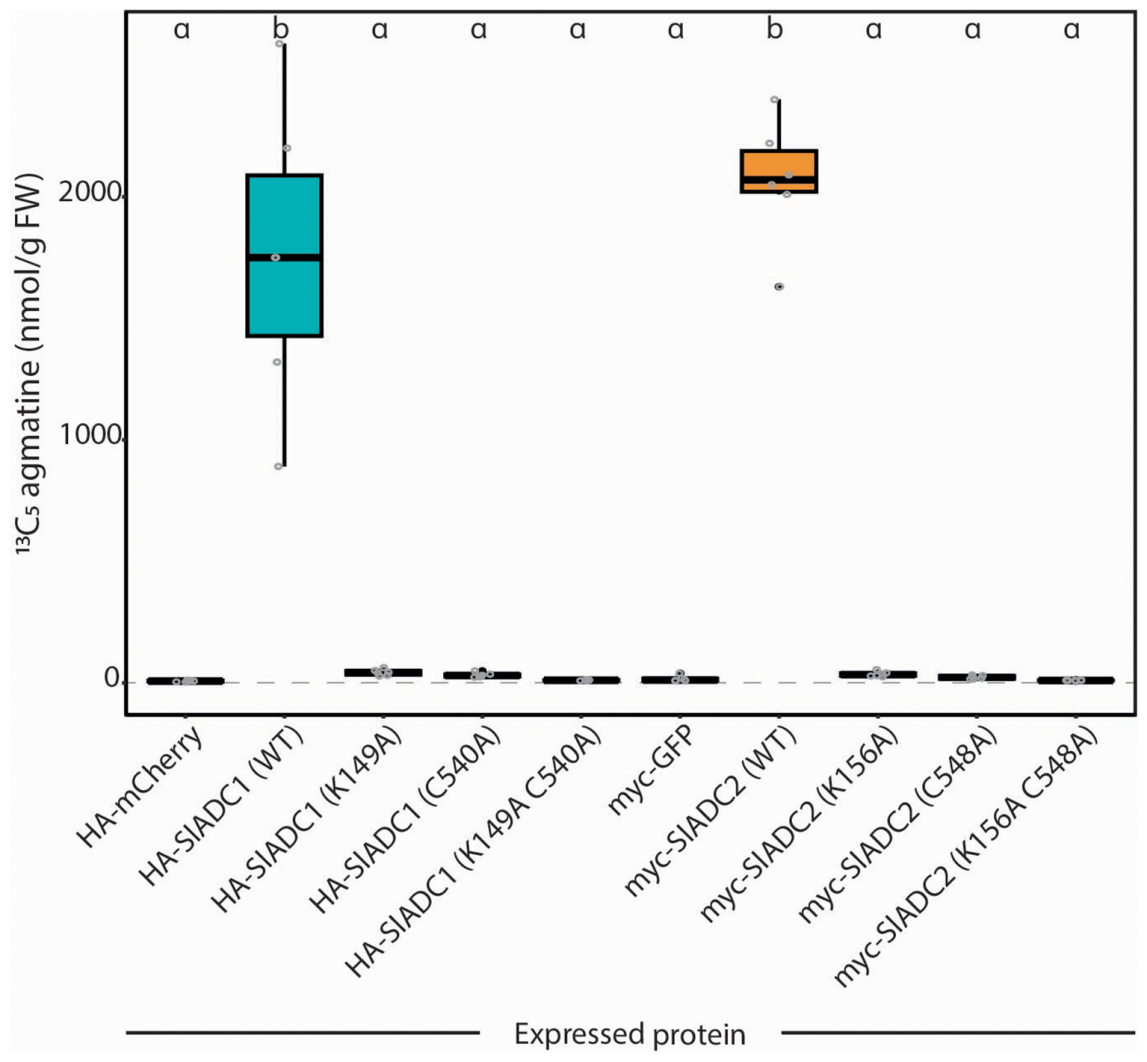
Mutating either the cofactor- or substrate-binding residues kills catalytic functionality of SlADC1 and SlADC2 *N. benthamiana* leaves were agroinfiltrated with 35S-promoter driven HA-SlADC1 (blue) or myc-SlADC2 (orange) WT or mutant variants, alongside HA-mCherry and myc-GFP fluorophores as negative controls. After 2dpi, ADC activity (^13^C_5_ agmatine in nmol/g FW, normalized to D_5_ tryptophan) was quantified using the optimized LC-MS method. Statistical significance determined using ANOVA followed by Tukey’s *post hoc* HSD test; different letters above each box indicate statistically significantly different groups (α = 0.05). Each biological replicate represented by a grey circle; n = 6.

We next wanted to evaluate whether the enzymatic functionality of ADC mutant variants, recombinantly expressed in *N. benthamiana* leaves, can be assessed using our optimized ADC activity assay. We generated *SlADC1* and *SlADC2* mutants in the key catalytic lysine and cystine residues that are essential for enzymatic function (**Supplementary Figure 6**). These residues were either individually mutated (i.e., SlADC1 K149A or C540A, and SlADC2 K156A or C548A) or both mutated to alanine (i.e., SlADC1 K149A C540A and SlADC2 K156A C548A). T-DNA constructs were generated encoding *35S*-promoter driven, epitope-tagged versions of mutated *SlADC1/2*, transformed into *A. tumefaciens*, and agroinfiltrated into *N. benthamiana* leaves alongside complementary mCherry and GFP fluorophores, serving as negative controls. LC-MS analysis of wild-type (WT) SlADC1- and SlADC2-agroinfiltrated samples showed 72- and 85-fold higher ADC activity compared to the average ADC activity of the negative controls (**Figure 5**). No significant difference between the negative controls and any single or double SlADC1 or SlADC2 mutant variant was found, despite all proteins being equally expressed (**Supplementary Figure 7**). These results demonstrate that both lysine and cysteine residues are essential for SlADC1/2 catalytic activity.

### Analyzing substrate specificity of transiently expressed ADC and ODC enzymes in *N. benthamiana*

As ADCs and ODCs are key enzymes in PA biosynthesis, we wanted to show that our LC-MS assay could quantify both activities following their transient expression in *N. benthamiana*. We generated *35S*-promoter driven T-DNAs encoding, HA epitope-tagged SlADC1, SlADC2, SlODC1, and mCherry (negative control) and agroinfiltrated these into *N. benthamiana* leaves. At 2 dpi, samples were harvested for both LC-MS and immunoblot analysis. Immunoblot analysis showed that all proteins were detectable (**Supplementary Figure 8**).

**Figure 6:**
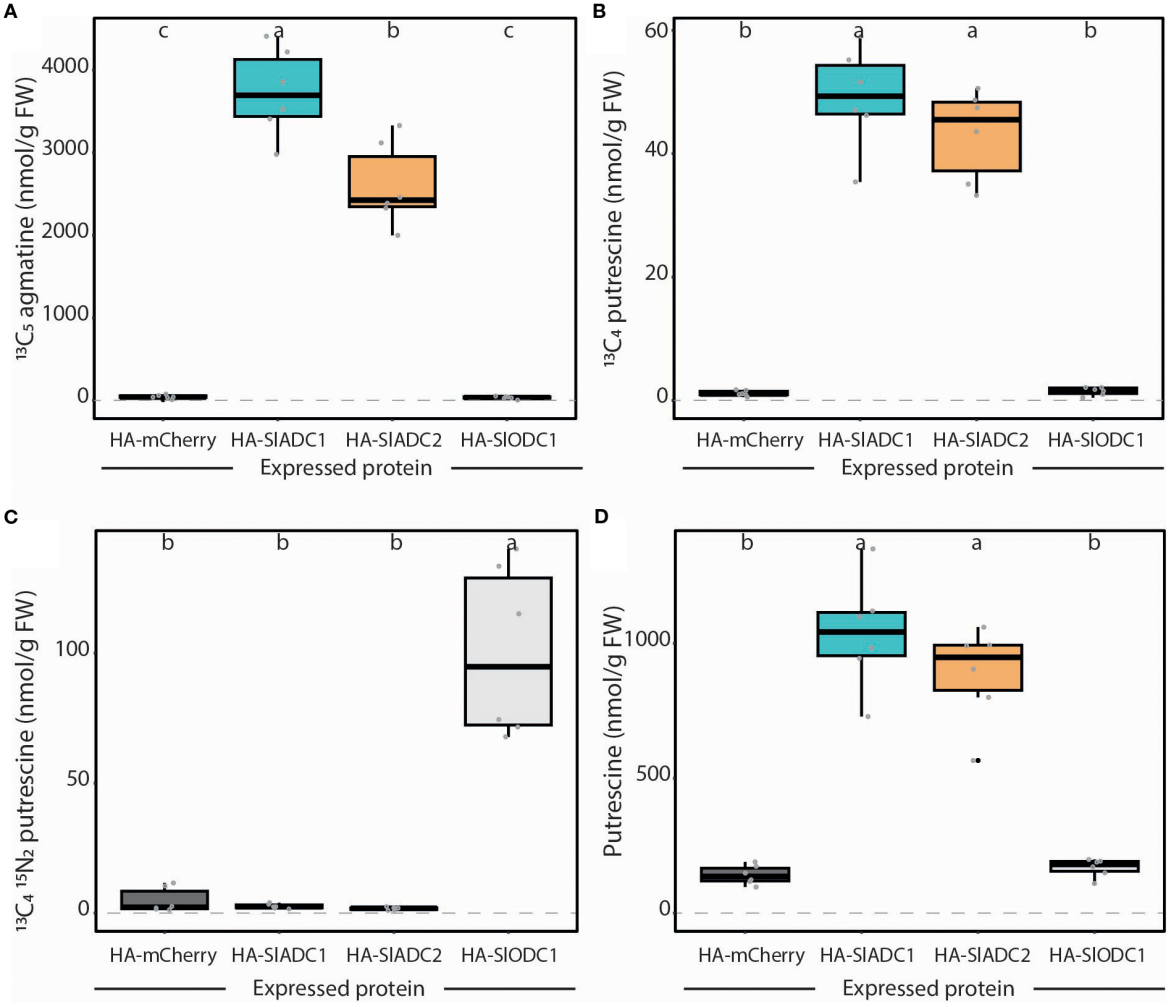
Transiently expressed tomato ADC and ODC enzymes show high substrate specificity. *N. benthamiana* leaves were agroinfiltrated with 35S-promoter driven HA epitope-tagged mCherry (dark grey), SlADC1 (blue), SlADC2 (orange), or SlODC1 (light grey) constructs. **(A)** ADC activity (^13^C_5_ agmatine) and **(B)** ^13^C_4_ putrescine can be detected following expression of tomato ADC enzymes. **(C)** Only expression of tomato ODC1 produced high ODC activity (^13^C_4_^15^N_2_ putrescine). **(D)** Expression of tomato ADCs, but not ODC1, induced putrescine levels. All metabolite levels are presented in nmol/g FW, normalized to D_5_ tryptophan. Statistical significance determined using ANOVA followed by Tukey’s *post hoc* HSD test; different letters above each box indicate statistically significantly different groups (α = 0.05). Each biological replicate represented by a grey circle; n = 6.

As anticipated, transient expression of SlADC1 and SlADC2 produced 83- and 58-fold higher ADC activity levels compared to the negative mCherry control, whilst SlODC1 expression produced no significant increase in ADC activity (**Figure 6A**). This pattern was mirrored in the accumulation of ^13^C_4_ putrescine (generated from ^13^C_6_ arginine), where SlADC1 and SlADC2 expression caused 42- and 43-fold higher levels compared to the control (**Figure 6B**). On the other hand, 20-fold higher ODC activity was observed upon transient SlODC1 expression, whilst no significant increase was observed following SlADC1 and SlADC2 expression (**Figure 6C**). Interestingly, expression of SlADC1 and SlADC2 enzymes, but not SlODC1, significantly increased putrescine compared to the mCherry control (**Figure 6D**). Also in this assay, we included a ^13^C_6_^15^N_2_ lysine substrate along with the isotopic variants of arginine and ornithine to determine substrate specificity of SlADC and SlODC. As expected, no LDC activity (i.e., production of ^13^C_5_^15^N_2_ cadaverine from ^13^C_6_^15^N_2_ lysine) was detected above background levels from any expressed enzyme.

Overall, these results demonstrate that tomato ADC and ODC enzymes have strict preference for their respective metabolic substrates and that their activities can be easily analyzed following transient expression in *N. benthamiana* leaves without the need for *in vitro* protein purification.

### Characterization of the PA network and phenotypes of tomato ADC and ODC knock out mutants

**Figure 7:**
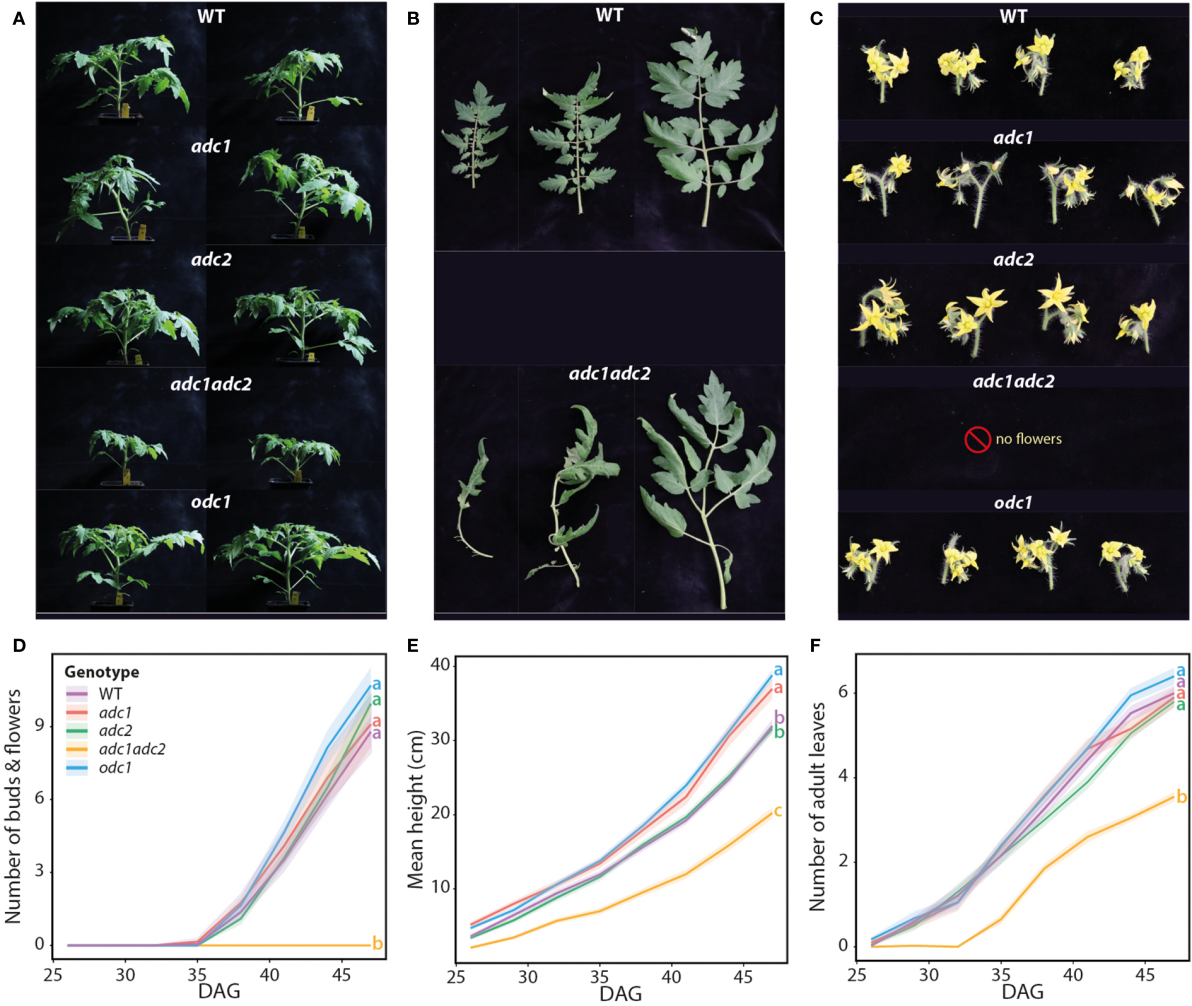
Tomato *adc1/adc2* mutant is smaller with fewer leaves and inability to form flowers. Phenotypes of WT, *adc1*, *adc2*, *adc1/adc2*, and *odc1* tomato mutants were analyzed until 47 days after germination (DAG). **(A)** Photos of whole plants were taken at 37 DAG; two representatives are shown. **(B)** Leaves of the *adc1/adc2* double mutant have morphological differences to WT. Photos taken 47 DAG. **(C)** At 47 DAG, photos were taken of flowers from each genotype, except for *adc1/adc2* as no flowers were ever formed. **(D)** Flower and bud numbers were counted. **(E)** Double *adc1/adc2* mutant plants were also shorter and **(F)** had fewer adult leaves. All plants (WT [purple], *adc1* [red], *adc2* [green], *adc1/adc2* [yellow], and *odc1* [blue]) were analyzed every 3 days from 26-47 DAG; n = 20. Solid lines represent mean and shaded area represents standard error of the mean. Statistical significance was determined only on data from 47 DAG; an ANOVA followed by Tukey’s *post hoc* HSD test was conducted; different letters above each box indicate statistically significantly different groups (α = 0.05).

To gain deeper insight into the PA network, we analyzed tomato *adc1*, *adc2*, and *adc1/adc2* mutants (Wu et al., 2019) using our optimized LC-MS profiling method. We also included an unpublished single *odc1* tomato mutant, with the remaining two *SlODC* genes WT (*Solyc03g098300.1* and *Solyc03g098310.1*). These tomato lines were all generated via CRISPR-Cas9 mutagenesis and carry deletions that lead to premature stop codons (Wu et al., 2019; **Supplementary Figure 9**). In contrast to *A. thaliana* where the loss of both *ADC* genes is embryonically lethal due to the absence of *ODC*, the tomato *adc1/adc2* double mutant is viable, but cannot produce seeds. Since *adc1/adc2* mutants produce no seeds, we identified these double mutants by genotyping the progeny of a parental line that was homozygous mutant for *SlADC1* (sl*adc1/sladc1*) and heterozygous for *SlADC2* (*SlADC2/sladc2*) (**Supplementary Figure 9D**). Since *adc1/adc2* mutants offer a unique opportunity to study the functional roles of ADCs, we analyzed phenotypic differences between these mutants and WT plants. Twenty genotyped tomato plants representing WT, *adc1*, *adc2*, *adc1/adc2*, and *odc1* were examined every three days between 26 to 47 days after germination (DAG), with photos taken of representative plants at 37 DAG (**Figure 7A**). Leaves of *adc1/adc2* mutants developed primary and secondary leaflets more slowly, often lacking intercalary leaflets entirely (**Figure 7B**). By 47 DAG, their leaves and leaflets were less flat and expanded compared to those of WT plants. Interestingly, no flowers, buds, or tissues indicating developing flower buds were ever observed on *adc1/adc2* plants, demonstrating that *ADC* genes are indispensable for flower development (**Figure 7C, D**). By contrast, the single *adc1* or *adc2* mutants showed no obvious deficiency in flower development compared to WT. Additionally, the double *adc1/adc2* mutant plants were shorter and had fewer adult leaves compared to WT or the other mutants (**Figures 7E, F**). Intriguingly, *adc1* and *odc1* were both significantly taller 47 DAG compared to the WT. Overall, this phenotypic analysis demonstrates the importance of ADCs for tomato growth and development of reproductive organs.

**Figure 8:**
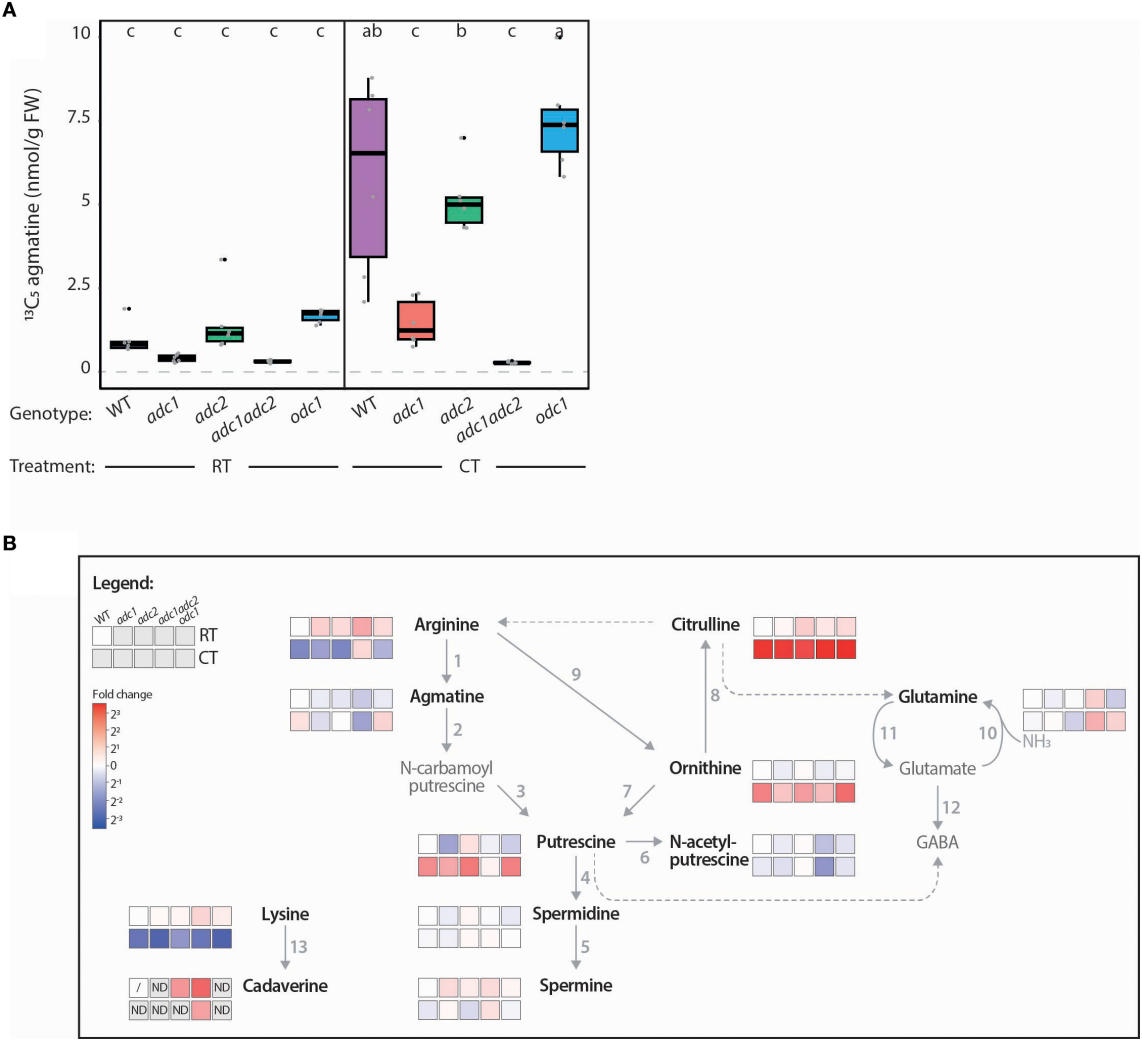
Tomato *adc/1adc2* mutant has no ADC activity and perturbed PA metabolism. **(A)** ADC activity of *adc1* and *adc1/adc2* is severely affected in both room temperature (RT) and cold treated (CT) conditions. ADC activity (^13^C_5_ agmatine in nmol/g FW, normalized to D_5_ tryptophan) of WT (purple), *adc1* (red), *adc2* (green), *adc1/adc2* (yellow), and *odc1* (blue) adult tomato leaves following RT or CT at 38 DAG. Each biological replicate represented by a grey circle (n = 6). Statistical significance determined using ANOVA followed by Tukey’s *post hoc* HSD test; different letters above each box indicate statistically significantly different groups (α = 0.05). **(B)** Heatmap pathway representation demonstrates impact of knocking out *ADC*s on PA metabolism in tomatoes. Prior to the assay incubation, 100 µL of sample was removed and immediately stopped with HFBA; this was analyzed by LC-MS. The log2 fold change (FC) of each metabolite was calculated relative to the RT-treated WT (WT RT) control; red indicates higher and blue lower metabolite levels compared to the WT RT. Solid arrows represent reactions catalyzed by a single enzyme, while dotted arrows indicate multiple enzymatic steps. If a metabolite was not detected (ND) the box is shaded grey. A metabolite not detected in all biological replicates of the WT RT reference is symbolized with a dash. Numbers correlate to the following enzymes: 1, ADC; 2, AIH; 3, NCPAH; 4, SPDS; 5, SPMS; 6, NATA1; 7, ODC; 8, OTC; 9, ARGAH; 10 GOGAT; 11, GS; 12, GAD; and 13, LDC. Only bold, black named metabolites were measured in this LC-MS experiment.

To determine the role each ADC enzyme plays in PA metabolism, transcript levels, ADC activity and PA-related metabolites were quantified in all five genotypes following an acute CT. *SlADC1* and *SlADC2* transcript levels were increased in all genotypes following CT, except for *SlADC1* in the *adc1* mutant (**Supplementary Figure 10**). ADC activity was detectable for all genotypes following RT or CT, except for the *adc1/adc2* double mutant (**Figure 8A**), consistent with agmatine being exclusively synthesized by ADCs. Following CT, ADC activity was significantly induced in the WT, *adc2*, and *odc1* genotypes, but not *adc1*, despite significant transcriptional activation of its functional *SlADC2* gene following CT (**Supplementary Figure 10B**). These results indicate that SlADC1 is the main contributor to ADC activity in adult tomato leaves following CT.

When quantifying PA metabolites, we again observed higher agmatine, putrescine, citrulline, and ornithine levels and lower arginine levels in WT tomato leaves following CT (**Figure 8B**). Contrastingly, the *adc1/adc2* double mutant had no detectable agmatine above background levels and under both RT and CT conditions the *adc1/adc2* mutant had higher arginine but lower putrescine levels. Interestingly, N-acetylputrescine levels were also lower in the *adc1/adc2* mutant compared to WT, while spermidine and spermine levels remained unaffected. Finally, we noted that although lysine (the cadaverine precursor) was lower in all genotypes following CT, cadaverine was not increased and was only reliably detected in the *adc1/adc2* mutant under both conditions.

In summary, we characterized the phenotypes, ADC activities, and PA network in the tomato *adc1*, *adc2*, *adc1/adc2*, and *odc1* mutants, demonstrating the importance of ADC enzymes not only for PA synthesis, but also for plant development and generation of reproductive tissues.

## Discussion

### Optimizations vastly improved the previous LC-MS-based ADC activity assay

In this work we optimized the sensitivity and workflow of our previously established LC-MS-based method for quantifying ADC activity and PAs from plant extracts. Moreover, we broadened the scope of this method, including ODC activity and 7 more PA-related metabolites (i.e., arginine, ornithine, citrulline, glutamine, N-acetylputrescine, lysine, and cadaverine) that were not covered by the previous method. Multiple aspects of our LC-MS approach were considered for optimization, including substrate concentration, extraction method, and presence of the ion-pairing agent HFBA in the solvent.

### Higher substrate concentration increases ADC activity levels

Using 10-fold higher substrate concentration increased ADC activity ~11-fold (**Figure 2C**), producing clear ^13^C_5_ agmatine LC-MS peaks well above detection limits, even from samples with low levels of ADC proteins (e.g., RT single *adc* mutants; **Figure 8**). The ^13^C_6_ arginine-derived putrescine variant (^13^C_4_ putrescine) could only be detected with elevated substrate levels, but not at lower ^13^C_6_ arginine concentrations (**Figure 2D**). Since at both substrate concentrations ^13^C_6_ arginine levels were never depleted (**Supplementary Data File**), the higher concentration (i.e., 500 µM) likely increased ADC activity by almost reaching the reported SlADC1 Km of 600 µM (Guo et al., 2023), a situation representing 50% substrate saturation of the enzyme. A higher ^13^C_6_ arginine concentration to achieve 100% enzyme substrate saturation was not considered as this could negatively affect the chromatographic separation and electrospray ionization process of coeluting metabolites (Jessome and Volmer, 2006). Taken together, our results suggest that 500 µM ^13^C_6_ arginine increased assay sensitivity, facilitating detection of subtle fluctuations in ADC activity that occur in response to abiotic stresses.

### Simultaneous quantification of ADC and ODC activity from plant tissue

Putrescine, the precursor for synthesizing higher plant PAs, is produced via the ADC- or ODC-dependent pathways. We aimed to establish an approach allowing quantification of both ADC and ODC enzymatic activities from the same tissue extract in one LC-MS sample. To quantify ODC activity, we used a stable ^13^C_5_^15^N_2_ ornithine isotope that, following decarboxylation by ODC, produces ^13^C_4_^15^N_2_ putrescine. This putrescine isotope differs in its molecular mass sufficiently from the ADC-derived ^13^C_4_ putrescine (i.e., from ^13^C_6_ arginine), enabling quantification of both ADC and ODC activities simultaneously (**Figure 2F**). In contrast to ADC activity, increasing ^13^C_5_^15^N_2_ ornithine substrate concentration did not elevate ODC activity. This is likely due to low SlODC protein levels as *SlODC* is not highly transcribed in adult tomato leaves (Kwak and Lee, 2001; Acosta et al., 2005; Sivakumar et al., 2022).

### ODC activity is higher in whole extracts containing all organelles

We compared ADC and ODC activities in WEs and SNs (**Figure 2B**) and consistently found higher ODC activity in WEs than SNs (**Figure 2F**). ODCs have been shown to localize to the endoplasmic reticulum (ER, Joshi et al., 2024) and predicted to localize to chloroplasts and mitochondria (Sivakumar et al., 2022). Centrifugation to obtain SNs removes these key organelles, thereby reducing ODC abundance and activity. In contrast, ADC activity did not differ between SNs and WEs (**Figure 2C**), indicating that SlADCs are predominantly localized in the cytoplasm of tomato leaf cells. Overall, this work demonstrates that analysis of WEs simplifies the workflow and is equally suitable for enzymes localized in the cytoplasm, ER or plastids. Comparative LC-MS analysis of SN and WE samples could also provide information regarding localization of natively expressed enzymes, thereby complementing classical subcellular localization studies.

### Additional HFBA in the LC-MS gradient is not required for high quality metabolite quantification

Finally, we tested whether HFBA could be omitted from the LC-MS gradient whilst still enabling reliable ADC activity quantification. HFBA was used for two purposes in the previously published method: firstly, to stop proteinaceous reactions (i.e., enzymatic activity) at a defined timepoint (**Figure 2B**); and secondly, as an ion pairing agent, that improves resolution of charged metabolites. However, HFBA potentially reduces LC-MS platform longevity and integrity, as well as creating matrix suppression effects. Upon comparing metabolite peak areas of the same samples analyzed with or without HFBA in the LC-MS gradient, we observed no significant differences (**Supplementary Figure 3**), indicating that sufficient ion-pairing occurs from the HFBA added directly into the samples. Therefore, HFBA can be omitted from the LC-MS gradient without compromising metabolite and enzyme quantification, improving LC-MS system longevity.

### Studying post-transcriptional regulation: ADC and ODC activity quantification as a proxy for their protein abundance *in planta*

A gene’s transcript level is commonly used to estimate the amount and biological activity of the encoded protein, yet this approach is unsuitable for ADC and ODC, whose translation is also regulated post-transcriptionally (Kwak and Lee, 2001; Wu et al., 2019; Jiménez-Bremont et al., 2022). Here, we validated a linear relationship between ADC activity and ADC protein abundance (**Figure 4**), showing that enzyme activity can be used as a proxy for protein abundance *in planta*. Therefore, with the simplicity and reliability of our LC-MS approach, it is now possible to efficiently compare ADC and ODC activities, and accordingly their protein levels, across many tissues and/or stimuli.

Furthermore, systematic comparison of *ADC*/*ODC* mRNA levels with enzyme activities quantified using our LC-MS approach could identify contexts where transcript levels and enzyme activities differ most. As such contexts with large differences would be indicative of post-transcriptional regulation, our approach has the potential to be used in systematic screens, uncovering tissue contexts or stimuli where ADC and ODC expression is regulated at the post-transcriptional level.

### Additional purification step opens the door for analysis of transiently expressed ADC and ODC enzymes

*Agrobacterium*-mediated transfer of T-DNAs encoding proteins of interest into *N. benthamiana* leaves has become a widely used method for functional analysis of plant genes. We therefore wanted to ensure that our LC-MS approach could be applied to ADC/ODC proteins ectopically expressed *N. benthamiana* leaves. Yet, when using the standard extraction protocol, these samples clogged our Micro-LC columns. This is likely due to high alkaloid levels in this species, making up to ~1% of its dry weight (Stephan et al., 2018), which would also be present in the WE samples. By including an additional extraction step, *N. benthamiana* samples no longer interfered with the columns, enabling key experiments to be conducted (**Figures 4**-**6**). Using this system, we not only verified that ADC activity has a linear relationship to ADC protein abundance (**Figure 4**) but also confirmed the functional relevance of key lysine and cysteine residues required for SlADC activity (**Figure 5**). We were also able to study substrate specificities of key enzymes in the PA network by simply adding stable isotope variants of arginine, ornithine, and lysine to plant extracts. These assays showed that transiently expressed tomato ADCs and ODC1 enzymes had the expected substrate specificities (**Figure 6**). This extraction enables analysis of key PA biosynthetic enzymes, ADC and ODC, by leveraging transient expression in *N. benthamiana*. This allows comparative studies of enzyme mutants or homologs, or treatment responses (e.g., inhibitor, hormone, or abiotic/biotic stress treatments) all within the same *N. benthamiana* leaf background, eliminating the need for time- and resource-intensive recombinant protein expression and purification, thereby streamlining and accelerating enzyme activity studies.

*N. benthamiana* is not only a commonly used system for *in planta* recombinant expression of proteins of interest but also serves as a host for the bacterial model pathogens *Pseudomonas* and *Xanthomonas*. The assays established here now enable the combination of transient expression of enzymes that alter PA homeostasis with infections by *Pseudomonas* or *Xanthomonas*, both of which have been shown to manipulate the PA pathway through injected toxins or effector proteins (Kim et al., 2013; Guo et al., 2023; Gerlin et al., 2021). Such assays, together with our MS-based platform for PA quantification, could provide a powerful tool to investigate how different PAs influence bacterial infection.

### Broadening the scope of quantified metabolites provides insights into the wider PA network

We also aimed to broaden our portfolio of PA-related metabolites that can be quantified by our LC-MS approach, particularly focusing on anabolic pathway components, many of which play important roles in plant stress responses (Joshi and Fernie, 2017; Hildebrandt, 2018; Batista-Silva et al., 2019; Moormann et al., 2022). We compared 11 PA network members in RT and CT tomato leaves, finding a CT-dependent reduction of arginine and lysine levels and increased agmatine, putrescine, citrulline, and ornithine levels (**Figures 2**, **8**). The arginine pool was likely depleted due to increased *SlADC1/2* expression and ADC activity following CT (**Supplementary Figures 2**, **10**, **Figures 2C**, **8A**), accumulating agmatine for putrescine synthesis, a metabolite known to mitigate cold stress (Cuevas et al., 2008; Chen et al., 2019; Upadhyay et al., 2020; González-Hernández et al., 2022). Interestingly, despite increased ADC activity, agmatine, and putrescine levels, we never observed CT-dependent changes in spermidine or spermine levels (**Figures 3**, **8**). Similar results have been previously reported (Alcázar et al., 2005, 2006; Wu et al., 2019; Gallas et al., 2024), indicating that spermidine and spermine levels are strictly regulated, possibly by PA-dependent translational regulation (Jiménez-Bremont et al., 2022). Displaying the data as a pathway heatmap highlights metabolites whose homeostasis may be prioritized (i.e., spermidine and spermine) or diverted to other metabolic pathways during stress responses (e.g., lysine). Together, quantification and heatmap visualization of the PA network offer comprehensive insight into pathway fluxes, revealing genotype- or condition-specific changes and key regulatory nodes.

### Insights into the roles of SlADCs on the PA network and plant development using CRISPR-Cas9-generated tomato mutants

We also used our optimized LC-MS-based approach to study the role of tomato ADC and ODC enzymes on ADC activity and PA metabolism following an acute CT using single *adc1* and *adc2* and double *adc1/adc2* tomato mutants (Wu et al., 2019) and a novel *odc1* mutant (**Supplementary Figure 9**). Importantly, we confirmed that SlADC1 and SlADC2 are the sole enzymes capable of synthesizing agmatine in tomato as no ADC activity was observed above background levels in the *adc1/adc2* double mutant (**Figure 8A**). Accordingly, compared to WT, this *adc1/adc2* mutant had higher arginine levels and lower agmatine and putrescine under both RT and CT conditions (**Figure 8B**). This demonstrates that in the absence of functional ADC enzymes, arginine builds up, and as agmatine cannot be produced, putrescine synthesis is reduced. As *adc1/adc2* plants have a functional ODC pathway but still lower putrescine levels compared to WT, this indicates that SlODCs cannot compensate for the loss of SlADCs. Surprisingly, no difference in spermidine or spermine levels were observed in the *adc1/adc2* mutant compared to WT in either RT or CT samples, despite reduced levels of their precursor putrescine (**Figure 8B**). This suggests that despite completely lacking the ADC-dependent pathway, spermidine and spermine levels are maintained, indicating a prioritization regulatory mechanism maintaining their levels at the expense of putrescine. Alternatively, PAs may be synthesized in other tissues with higher ODC expression, such as the roots (Kwak and Lee, 2001; Acosta et al., 2005) and transported through the plant. Grafting the arial part of *adc1/adc2* plants to WT root stocks and analyzing ADC activity and PA levels in their leaves could give insight into long distance transport of such metabolites.

The *adc1* single mutant also had severely reduced ADC activity compared to *adc2* and WT in both RT and CT conditions (**Figure 8A**), indicating that ADC1 is the main contributor of ADC activity in tomato leaves. This is likely due to higher *SlADC1* expression in tomato leaves (**Supplementary Figures 2**, **10**), rather than SlADC1 being a more efficient enzyme, as we observed only slightly higher ADC activity levels when transiently overexpressing SlADC1 compared to SlADC2 in *N. benthamiana* (**Figures 4**-**6**).

We also characterized the phenotypes of these single and double tomato mutants, as a double *adc1/adc2* mutant in *A. thaliana* cannot be generated (Urano et al., 2005). Compared to WT, *adc1/adc2* mutant plants were severely affected, being only two-thirds as tall as the WT at 47 DAG, with significantly fewer adult leaves and no flowers (**Figure 7**). In fact, we never observed any tissue resembling developing floral buds on *adc1/adc2* plants, even after months of growth. Previous studies have found higher PA levels in floral tissues compared to leaves in various plant species and that supplementation of PAs can promote floral development (Chen et al., 2019). Spermine is essential for *A. thaliana* flower development (Zierer et al., 2016), yet no significant differences in spermidine or spermine levels were observed in *adc1/adc2* leaves compared to WT (**Figure 8**). On the other hand, putrescine levels, which were significantly reduced in *adc1/adc2* leaves, were reportedly highest in the early tomato flower stages (Antognoni et al., 2002). Overall, this work demonstrates that ADCs are essential for plant and flower development, even though the concentrations of key PAs, such as spermidine and spermine remain unchanged in the *adc1/adc2* mutant.

## Summary

In summary, we optimized our LC-MS-based method to quantify ADC and ODC enzyme activities and 11 PA-related metabolites in a single plant extract by a single MS run. This method has multiple advantages over other published methods: it is reasonably high throughput (manageable to extract and run up to 100 samples/day), less damaging to LC-MS systems (HFBA is not required in the LC-MS gradient), and does not require derivatization, reducing the overall cost and labor efforts of such an experiment. Furthermore, it is the only published method that measures both ADC and ODC enzyme activity and PA levels from the same sample. We believe our LC-MS-based method will be invaluable to researchers studying plant PA metabolism, including those investigating how plant pathogens manipulate this network via injected effectors or toxins (Wu et al., 2019; Gerlin et al., 2021), as well as those studying PA metabolism in species other than tomato or *N. benthamiana* or in other organisms. Given that ADCs are indispensable for plant reproduction and PAs play key roles in stress responses, our method could also be instrumental in advancing research areas across various fields of plant biology.

## Data Availability

The datasets presented in this study can be found in online repositories. The names of the repository/repositories and accession number(s) can be found in the article/**Supplementary Material**.
