# Supplementary material for: Optimized LC-MS method for simultaneous polyamine profiling and ADC/ODC activity quantification and evidence that ADCs are indispensable for flower development in tomato
Source: Front Plant Sci. 2025 Sep 11;16:1636076. doi: 10.3389/fpls.2025.1636076 (PMC12460301; doi:10.3389/fpls.2025.1636076)
Supplement: Supplementary file 1 [file Table1.docx]

| **Supplementary Table 1: Primers used in this work** | | | |
| --- | --- | --- | --- |
| Primer name | Primer sequence (5’ 🡪 3’) | Description | Origin |
| *SlODC1* F(1*) | CCGTCTCAAAGGGAACAGAGA | Screen for *Slodc1* CRISPR mutants | This study |
| *SlODC1* R (2*) | CTTGAGCTGTACGGAGGAGC | Screen for *Slodc1* CRISPR mutants | This study |
| *SlADC1* F (3*) | TACAGATACCCTTCCTCACCA | Screen for *Sladc1* CRISPR mutants | Wu et al., 2019 |
| *SlADC1* R (4*) | TGTCAATCACCAAATCAAGC | Screen for *Sladc1* CRISPR mutants | Wu et al., 2019 |
| *SlADC2* F (5*) | AATCGGCTTTTGACATGGCG | Screen for *Sladc2* CRISPR mutants | Wu et al., 2019 |
| *SlADC2* R (6*) | AAGGAGCTTTCTTGCGACCA | Screen for *Sladc2* CRISPR mutants | Wu et al., 2019 |
| qSlADC1 F | TAGCGAAAGTGGCAGAGCAA | qPCR for *SlADC1* in tomato | Wu et al., 2019 |
| qSlADC1 R | AGGTATCGTACTCTCCGCGA | qPCR for *SlADC1* in tomato | Wu et al., 2019 |
| qSlADC2 F | TTGGTCGCAAGAAAGCTCCT | qPCR for *SlADC2* in tomato | Wu et al., 2019 |
| qSlADC2 R | TGGCCAGAATGCTTTGTCCT | qPCR for *SlADC2* in tomato | Wu et al., 2019 |
| qTIP41 F | GCTTAGGGTTGATGGAGTGC | qPCR in tomato (Reference gene) | Wu et al., 2019 |
| qTIP41 R | CTCTCCAGCAGCTTTCACG | qPCR in tomato (Reference gene) | Wu et al., 2019 |
| SlADC1 BsaIC F | GGGTCTCACACCATGCCTGCTTTAGGTTGT | Clone *SlADC1* with BsaI cloning sites | This study |
| SlADC1 BsaID R | GGGTCTCACCTTCAAGCAACGCAGTAT | Clone *SlADC1* with BsaI cloning sites | This study |
| SlADC2 BsaIC F | GGGTCTCACACCATGCCGGCCTTAGGTTG | Clone *SlADC2* with BsaI cloning sites | This study |
| SlADC2 BsaID R | GGGTCTCACCTTAGTAGGACCAAATCTCATCTT | Clone *SlADC2* with BsaI cloning sites | This study |
| SlODC1 BsaIC F | TGGTCTCACACCATGGCCGGCCAAACAGT | Clone *SlODC1* with BsaI cloning sites | This study |
| SlODC1 BsaIC F | TGGTCTCACCTTGTTTGGATAAGCATAAGCAAGG | Clone *SlODC1* with BsaI cloning sites | This study |
| SlADC1 K149A F | ATGCAATCAGGACAGGTTC | Mutate SlADC1 (K149A) | This study |
| SlADC1 K149A R | GCTACAGGATAAACACCTTGGTA | Mutate SlADC1 (K149A) | This study |
| SlADC1 C540A F | TGACAGTGATGGAAAGATCG | Mutate SlADC1 (C540A) | This study |
| SlADC1 C540A R | GCTGTCAAGTCGGATAGAATTCC | Mutate SlADC1 (C540A) | This study |
| SlADC2 K156A F | ATGCAATCAAGATAGGTTCGT | Mutate SlADC2 (K156A) | This study |
| SlADC2 K156A R | GCCACCGGATAAACACCTTG | Mutate SlADC2 (K156A) | This study |
| SlADC2 C548A F | TGACAGTGATGGAAAGGTTG | Mutate SlADC2 (C548A) | This study |
| SlADC2 C548A R | GCCGTCAGGTCAGACAGTATT | Mutate SlADC2 (C548A) | This study |
| * Primer numbers in Supplementary Figure 8  F or R refer to forward or reverse primers, respectively  BsaIC/D refer to addition of BsaI restriction enzyme recognition sequences with overhangs (C or D) of Golden Gate toolkit (Binder et al., 2014) | | | |

# Supplementary tables:

| **Supplementary Table 2: Transitions (Q1/Q3), declustering potential (DP), collision energy (CE), retention times (RT), limit of detection (LOD), limit of quantitation (LOQ) and the linear range for the recorded analytes** | | | | | | | | |
| --- | --- | --- | --- | --- | --- | --- | --- | --- |
| Q1 mass (Da) | Q3 mass (Da) | DP  (volts) | CE  (volts) | Compound | RT  min | LOD  nM | LOQ  nM | Linear range  nM |
| 175.1 | 158.0 | 50 | 20 | arginine 1 | 2.47 |  |  |  |
| **175.1** | **116.1** | 50 | 20 | arginine 2 |  | 0.1 | 1 | 1-10 |
| 181.1 | 164.1 | 50 | 20 | ^13^C_6_ arginine 1 | 2.47 |  |  |  |
| **181.1** | **121.1** | 50 | 20 | ^13^C_6_ arginine 2 |  |  |  |  |
| **182.1** | **122.1** | 50 | 20 | ^13^C_6_ arginine 2 second isotopes * | 2.47 |  |  |  |
| 131.1 | 114.1 | 22 | 15 | agmatine 1 | 2.49 | 0.5 | 5 | 5-50 |
| **131.1** | **72.1** | 22 | 20 | agmatine 2 |  |  |  |  |
| 136.1 | 119.1 | 22 | 15 | ^13^C_5_ agmatine 1 | 2.49 |  |  |  |
| **136.1** | **76.1** | 22 | 20 | ^13^C_5_ agmatine 2 |  |  |  |  |
| 133.0 | 116.0 | 50 | 10 | ornithine 1 | 2.40 |  |  |  |
| **133.0** | **70.0** | 50 | 20 | ornithine 2 |  | 0.1 | 1 | 1-100 |
| 140.1 | 122.1 | 50 | 10 | ^13^C_5_^15^N_2_ ornithine 1 | 2.40 |  |  |  |
| **140.1** | **75.0** | 50 | 20 | ^13^C_5_^15^N_2_ ornithine 2 |  |  |  |  |
| **141.1** | **76.0** | 50 | 20 | ^13^C_5_^15^N_2_ ornithine 2 second isotopes * | 2.40 |  |  |  |
| **89.1** | **72** | 22 | 14 | putrescine 1 | 2.45 | 1 | 1 | 5-100 |
| 89.1 | 72 | 22 | 25 | putrescine 2 |  |  |  |  |
| **93.1** | **76.1** | 22 | 14 | ^13^C_4_ putrescine | 2.45 |  |  |  |
| **95.1** | **77.1** | 22 | 14 | ^13^C_4_^15^N_2_ putrescine | 2.45 |  |  |  |
| **131.1** | **114.1** | 22 | 15 | N-acetylputrescine 1 | 2.18 | 0.5 | 1 | 1-100 |
| 131.1 | 72.0 | 22 | 20 | N-acetylputrescine 2 |  |  |  |  |
| 146.15 | 129 | 20 | 20 | spermidine 1 | 2.51 |  |  |  |
| **146.15** | **72** | 20 | 20 | spermidine 2 |  | 0.1 | 1 | 1-50 |
| 203.2 | 112 | 48 | 15 | spermine 1 | 2.58 |  |  |  |
| **203.2** | **129.1** | 48 | 24 | spermine 2 |  | 0.1 | 10 | 10-250 |
| **176.1** | **159.1** | 22 | 15 | citrulline 1 | 1.84 | 0.1 | 0.5 | 0.5-250 |
| 176.1 | 113.1 | 22 | 25 | citrulline 2 |  |  |  |  |
| **179.1** | **162.1** | 22 | 15 | ^13^C_1_D_2_ citrulline 1 | 1.84 | 0.1 | 0.5 | 0.5-250 |
| 179.1 | 116.2 | 22 | 20 | ^13^C_1_D_2_ citrulline 2 |  |  |  |  |
| 147.1 | 130.0 | 50 | 15 | lysine 1 | 2.42 |  |  |  |
| **147.1** | **84.0** | 50 | 25 | lysine 2 |  | 1 | 1 | 1-50 |
| **156.1** | **137.1** | 50 | 15 | ^13^C_6_^15^N_2_ lysine 2 second isotopes * | 2.42 |  |  |  |
| **103.1** | **86.0** | 40 | 25 | cadaverine 1 | 2.45 | 0.1 | 0.1 | 0.1-10 |
| 103.1 | 69.1 | 40 | 25 | cadaverine 2 |  |  |  |  |
| **110.1** | **92.0** | 40 | 15 | ^13^C_5_^15^N_2_ cadaverine 1 | 2.45 |  |  |  |
| 110.1 | 74.1 | 40 | 25 | ^13^C_5_^15^N_2_ cadaverine 2 |  |  |  |  |
| **147.1** | **130.0** | 40 | 10 | glutamine 1 | 1.80 | 10 | 10 | 10-500 |
| 147.1 | 84.0 | 40 | 30 | glutamine 2 |  |  |  |  |
| **104.1** | **87.0** | 90 | 17 | γ-aminobutyric acid (GABA) 1 | 1.84 | 10 | 10 | 10-500 |
| **104.1** | **86.0** | 90 | 13 | β-aminobutyric acid (BABA) 1 | 2.24 |  |  |  |
| **104.1** | **58.0** | 90 | 19 | α-aminobutyric acid (AABA) 1 | 2.12 |  |  |  |
| **138.1** | **121.1** | 40 | 15 | tyramine 1 | 2.45 | 0.1 | 1 | 1.50 |
| 138.1 | 103.1 | 40 | 30 | tyramine 2 |  |  |  |  |
| 210.1 | 192 | 60 | 22 | D_5_ tryptophane 1 | 2.56 |  |  |  |
| **192.1** | **150.1** | 100 | 22 | D_5_ tryptophane 2 |  | 0.1 | 0.1 | 0.1-50 |
| Multiple reaction monitoring (MRM) transitions in bold letters represent the quantifier ions. Limits of detection (LOD) and limits of quantitation (LOQ) were determined based on visual evaluation, signal‑to‑noise (S/N) determination, and on the standard deviation of the response and calibration curve slope. S/N was calculated using the relative noise and autopeak integration algorithm of the Sciex OS software. For evaluating the linear range of the calibration curves, a mastermix containing the listed compounds above (with the exception of ^13^C_5_ agmatine, ^13^C_4_ putrescine, ^13^C_4_^15^N_2_ putrescine, and ^13^C_5_^15^N_2_ cadaverine) was diluted accordingly in the range of the LOD and LOQ in water with 0.1% HFBA and 0.1% FA. * The second isotope transitions of ^13^C_6_ arginine, ^13^C_5_^15^N_2_ ornithine, and ^13^C_6_^15^N_2_ lysine were only monitored to check for substrate presence in the enzyme assays. The lower response pairs were chosen to prevent detector blinding of the MS system while using substrate levels in the µM range. | | | | | | | | |

| **Supplementary Table 3: Chromatographic separation and LC‑MS settings** | | |
| --- | --- | --- |
| time: **main separation gradient** | % solvent A  (water, 0.1% aq. FA, + / - 0.05% HFBA) | % solvent B  (acetonitrile, 0.1% FA, + / - 0.05% HFBA) |
| 0 | 85 | 15 |
| 0.2 | 85 | 15 |
| 1.5 | 30 | 70 |
| 2.5 | 5 | 95 |
| 3 | 5 | 95 |
| 4 | 85 | 15 |
| 5 | 85 | 85 |
| time: **trap gradient** - **HFBA** | % solvent A  (water, 0.1% aq. FA) | % solvent B  (acetonitrile, 0.1% FA) |
| 0 | 98 | 2 |
| 1.8 change to separation gradient | 98 | 2 |
| 4 | 98 | 2 |
| time: **trap gradient + HFBA** | % solvent A  (water, 0.1% aq. (FA), 0.05% HFBA) | % solvent B  (acetonitrile, 0.1% FA, 0.05% HFBA |
| 0 | 98 | 2 |
| 2.5 change to separation gradient | 98 | 2 |
| 2.7 | 98 | 2 |
| Flow rate 16 µl/min for the separation gradient, 25 µl/min for the trap gradients. Column temperature 50C. MS settings: Optiflow Turbo V ion source with SteadySpray T micro electrode (10–50 μl/min); ion spray voltage: +4800 V; nebuliser, heater gas = nitrogen, 25 and 45 psi; curtain gas, nitrogen, 30 psi; collision gas, nitrogen, medium; source temperature, 200 °C; entrance potential, ±10 V; collision cell exit potential, ±10 V; dwell time 5 ms. | | |
