# Supplementary material for: Optimized LC-MS method for simultaneous polyamine profiling and ADC/ODC activity quantification and evidence that ADCs are indispensable for flower development in tomato
Source: Front Plant Sci. 2025 Sep 11;16:1636076. doi: 10.3389/fpls.2025.1636076 (PMC12460301; doi:10.3389/fpls.2025.1636076)
Supplement: Supplementary file 2 [file DataSheet1.pdf]

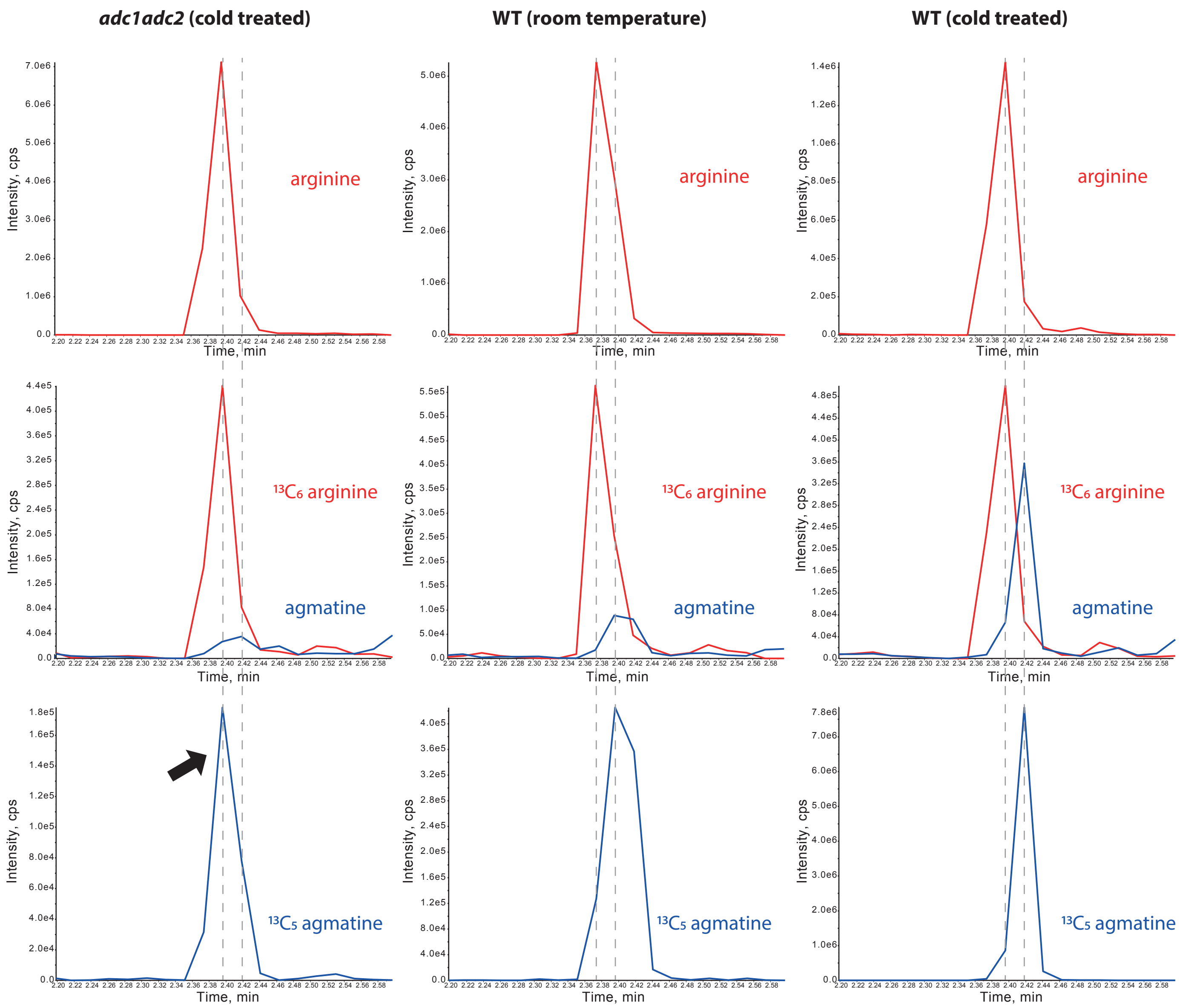

Supplementary Figure 1. Extracted ion chromatograms (XICs) of $^{13}C_5$ agmatine in *adc1/adc2* following cold treatment confirm lack of ADC activity.

XICs of cold treated (CT) *adc1/adc2* double mutant extracts (left column) showed only traces of $^{13}C_5$ agmatine (bottom row; black arrow), but this peak was congruent to arginine (red; top row) and $^{13}C_6$ arginine peaks (red; middle row; measured on the second isotope). Room temperature (RT) and CT treated WT plant extracts (middle and right column) show enzymatically produced $^{13}C_5$ agmatine (blue), which has a peak lining up to agmatine peaks (blue; middle panel). Dotted grey lines indicate either arginine or agmatine peaks in each plant extract.

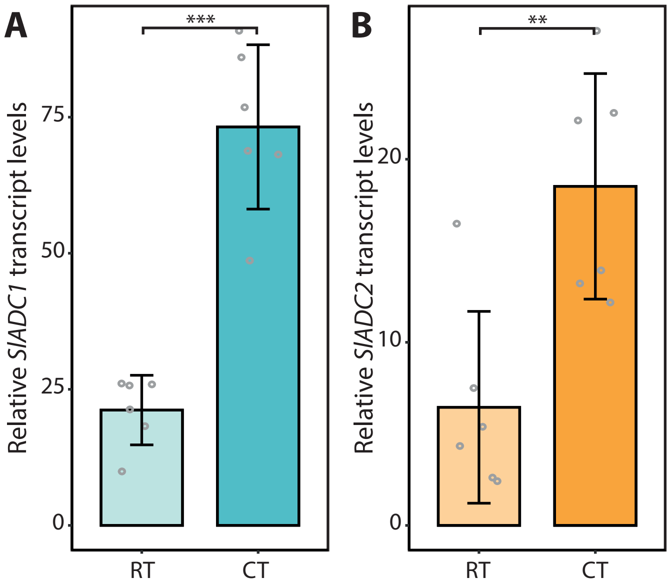

Supplementary Figure 2: Cold treatment of WT adult tomato leaves induces SlADC1 and SlADC2 expression.

RT-qPCR conducted on RNA extracted from 24-hour cold treated (CT) or room temperature (RT) control WT adult tomato leaves determining **A)** *SlADC1* and **B)** *SlADC2* transcript levels relative to the house keeping SlTIP41. Grey circles represent the mean of 3 technical replicates originating from one biological replicate (n =6). Statistical significance determined using a Student's T Test (a = 0.05); * = P-value < 0.05, ** = P-value < 0.01, and *** = P-value < 0.001.

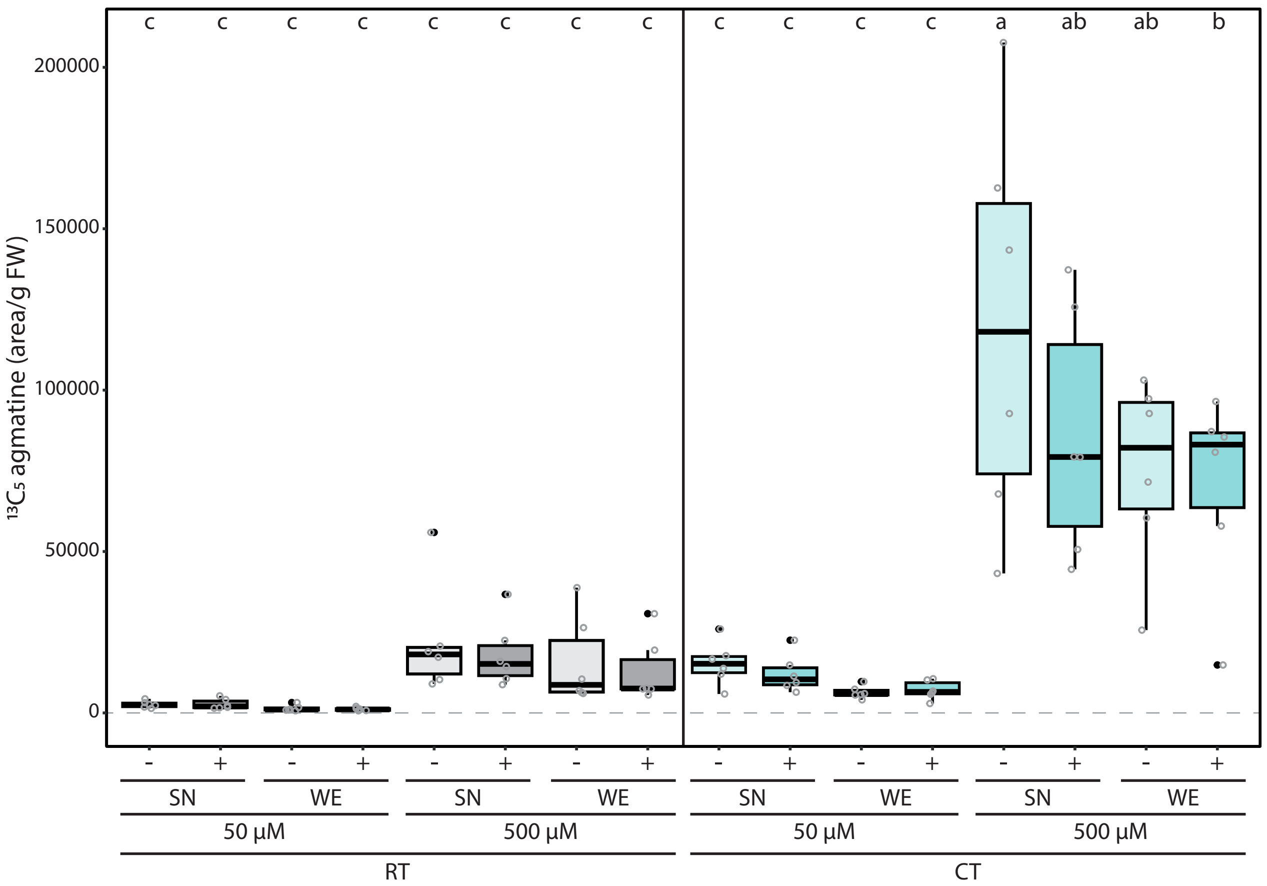

Supplementary Figure 3: Inclusion of HFBA in LC-MS gradient does not affect ADC activity quantification.

The same samples presented in Figure 2 that were run without HFBA in the LC-MS gradient solvent (-; light shades) were re-run with 0.1% HFBA included in the gradient (+; darker shades). ADC activity ($^{13}C_5$ agmatine in peak area/g fresh wight [FW] tissue, normalized to internal D_5 tryptophan standard) in tomato leaf samples, pretreated either with room temperature (RT; grey) or cold treatment (CT; blue), with either 50 or 500 µM $^{13}C_6$ arginine added to the SN or WE. Statistical significance determined using ANOVA followed by Tukey's post hoc HSD test; different letters above each box indicate statistically significantly groups (a = 0.05). Each biological replicate represented by a grey circle; n = 6.

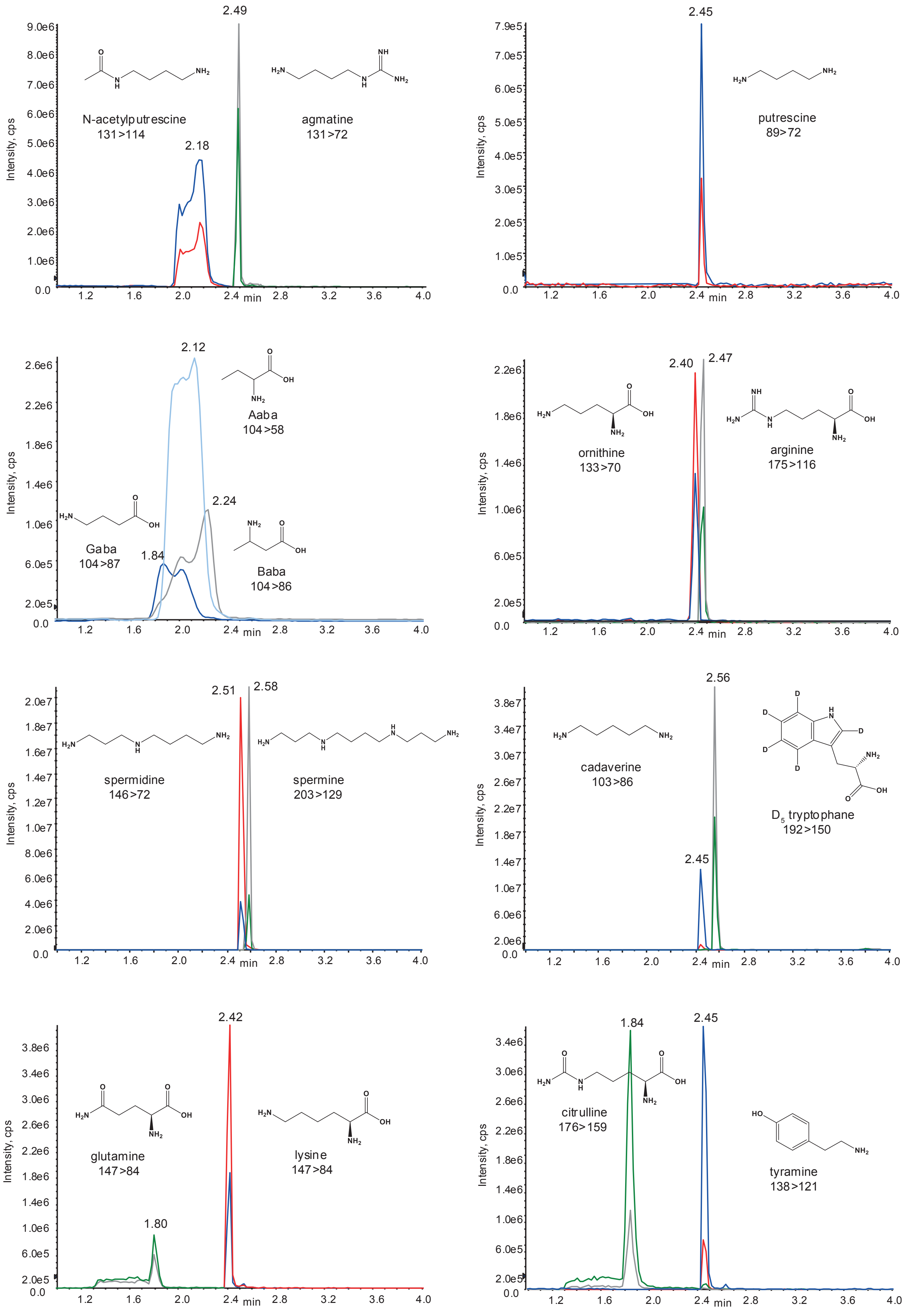

N-acetylputrescine
131>114
agmatine
131>72
2.49
2.18
putrescine
89>72
2.45
Gaba
104>87
Aaba
104>58
Baba
104>86
2.12
2.24
1.84
ornithine
133>70
arginine
175>116
2.40
2.47
spermidine
146>72
spermine
203>129
2.51
2.58
cadaverine
103>86
D5 tryptophane
192>150
2.56
2.45
glutamine
147>84
lysine
147>84
2.42
1.80
citrulline
176>159
tyramine
138>121
1.84
2.45
Intensity, cps
min

Supplementary Figure 4: Extracted ion chromatograms (XICs) of members of the PA network.

Zoomed in XICs (1-4 minutes of the 5 min gradient; counts per second [cps]), retention times (RT; min), and chemical structures of N-acetylputrescine, agmatine, putrescine, Gaba, Aaba, Baba, ornithine, arginine, spermidine, spermine, cadaverine, D_5 tryptophane, glutamine, lysine, citrulline, and tyramine. All standards were run together in a concentration of 100 nM, except the aminobutyric acid variants (1000 nM) and glutamine (200 nM), diluted in water with 0.1% formic acid and 0.1% HFBA. In most cases two MRM transitions are shown with the quantifier ion stated in the chromatogram (red or grey XIC). Additional information (LOQ, linearity range, qualifier ion transitions) can be found in Supplementary Table 1.

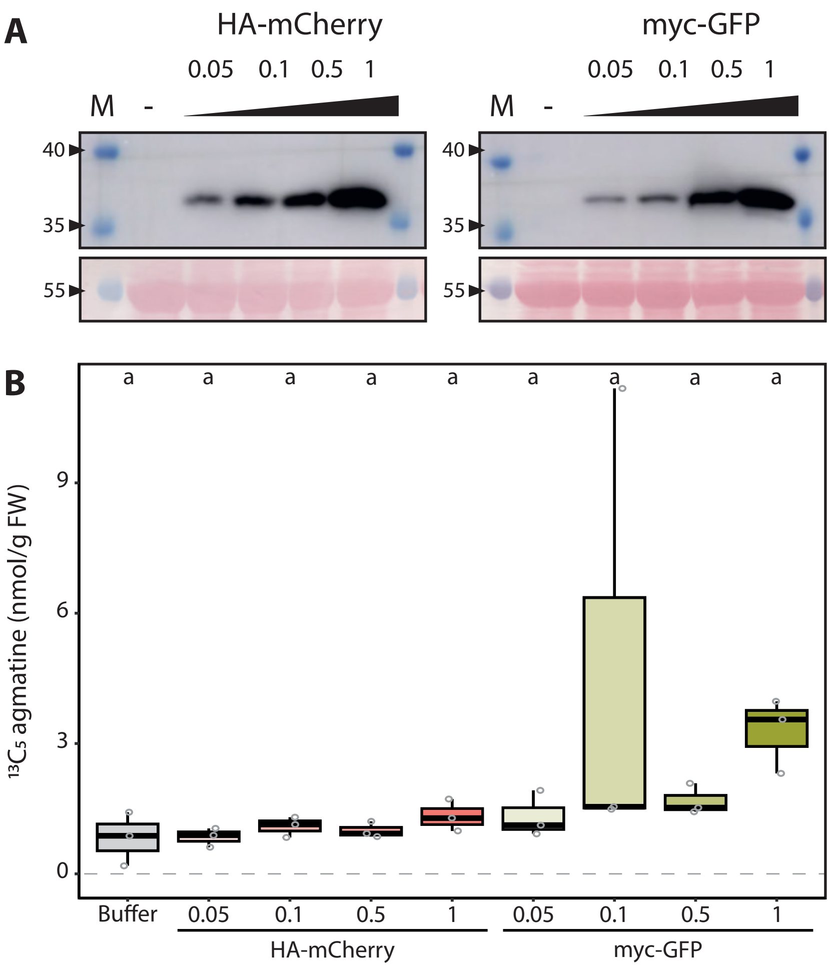

Supplementary Figure 5: Increasing amounts of fluorophore negative controls do not linearly correlate with rise in ADC activity.

A) Protein abundance of fluorophore controls increases with increasing bacterial load. Immunoblot analysis of *N. benthamiana* leaves agroinfiltrated with 35Sp:HA-mCherry:35St (left) and 35Sp:myc-GFP:35St (right) at increasing OD_{600} (0.05, 0.1, 0.5, and 1) of samples taken 2 days post infiltration (dpi). Samples of leaves infiltrated with only infiltration buffer were included as a negative control (-). Upper images (cropped) show HA-mCherry detection (left) and myc-GFP (right); expected size for both proteins is 29 kDa. Ponceau stained blots show equal protein loading (bottom). **B)** ADC activity does not correlate with increasing fluorophore protein abundance. ADC activity ($^{13}C_5$ agmatine in nmol/g FW, normalized to D_5 tryptophan) from the same *N. benthamiana* sample agroinfiltrated with HA-mCherry (red) or myc-GFP (green), or infiltration buffer only (grey) as shown in **A)**. Increasing color intensity represents increasing bacterial loads. Statistical significance determined using ANOVA followed by Tukey's post hoc HSD test; different letters above each box indicate statistically significantly different groups (a = 0.05 level). Each biological replicate represented by a grey circle; n = 3.

```
SlADC1          MPALGCCVESAVSPPLGYSFLST-----PEIFSSGVPPSTNAVPLTT--HWSPELSSDLY
SlADC2          MPALGCCVDASVSPPLGYAFSWDSSLPAPELFSSGVPPATNAAAVSTGSHWSTDLSSDLY
AtADC1          MPAL-----------------AFVDTPIDTFSSIFTPSSVSTAVVDGSCHWSPSLSSSLY
                ****                          : *  .*.:..:. :    ***..***.**

SlADC1          RIDGWGAPYFTVNSSGDISVRPHGTDTLPHQEIDLLKVVKKASDPINSGGLGLQLPLVVR
SlADC2          RVDGWGAPYFSVNSSGDISVRPHGTDTLPHQEIDLLKVVKKASDPKNLGGLGLQMPLVVR
AtADC1          RIDGWGAPYFAANSSGNISVRPHGSNTLPHQDIDLMKVVKKVTDP---SGLGLQLPLIVR
                *:********:.****:*******::*****:***:*****.:**   .*****:**:**

SlADC1          FPDVLKNRLESLQSAFDYAVQSEGYEAHYQGVYPVKCNQDRFVVEDIVKFGTGFRFGLEA
SlADC2          FPDVLKNRLETLQSAFDMAINSQGYEAHYQGVYPVKCNQDRFVVEDIVKFGSPYRFGLEA
AtADC1          FPDVLKNRLECLQSAFDYAIQSQGYDSHYQGVYPVKCNQDRFIIEDIVEFGSGFRFGLEA
                ********** ****** *::*:**::***************::****:**: :******

SlADC1          GSKPELLLAMSSLCKGSSEGLLVCNGFKDAEYISLALVARKLQLNTVIVLEQEEELDLVI
SlADC2          GSKPELLLAMNCLSKGSADALLVCNGFKDTEYISLALVARKLLLNSVIVLEQEEELDLVI
AtADC1          GSKPEILLAMSCLCKGNPEAFLVCNGFKDSEYISLALFGRKLELNTVIVLEQEEELDLVI
                *****:****..*.**..:.:********:*******..*** **:**************

SlADC1          DISRKMAVQPVIGLRAKLRTKHSGHFGSTSGEKGKFGLTTTQILRVVRKLKESGMLDCLQ
SlADC2          DISRKMSVRPVIGLRAKLRTKHSGHFGSTSGEKGKFGLTTTQILRVVKKLDESGMLDCLQ
AtADC1          DLSQKMNVRPVIGLRAKLRTKHSGHFGSTSGEKGKFGLTTVQILRVVRKLSQVGMLDCLQ
                *:*:** *:*******************************.******:**.: *******

SlADC1          LLHFHIGSQIPSTELLADGVGEAAQVYSELVRLGAGMKFIDIGGGLGIDYDGTKSSDSDV
SlADC2          LLHFHIGSQIPTTELLADGVGEATQIYSELVRLGAGMKFIDIGGGLGIDYDGSKSSNSDV
AtADC1          LLHFHIGSQIPSTALLSDGVAEAAQLYCELVRLGAHMKVIDIGGGLGIDYDGSKSGESDL
                ***********:* **:***.**:*:*.******* **.*************:**.:**:

SlADC1          SVGYGLQDYASTVVQAVRFVCDRKNVKHPVICSESGRAIVSHHSVLIFEAVSSTTTRS-Q
SlADC2          SVCYSIEEYASAVVQAVLYVCDRKGVKHPVICSESGRAIVSHHSILIFEAVSASTSHVST
AtADC1          SVAYSLEEYAAAVVASVRFVCDQKSVKHPVICSESGRAIVSHHSVLIFEAVSAG----QQ
                ** *.:::**::** :* :***:*.*******************:*******:

SlADC1          ELSSMSLHSFVEKLNDDARGDYRNLSAAAIRGEYDTCMLYADQLKQRCVDQFKDGNLDIE
SlADC2          QPSSGGLQSLVETLNEDARADYRNLSAAAVRGEYDTCLIYSDQLKQRCVEQFKDGSLDIE
AtADC1          HETPTDHQFMLEGYSEEVRGDYENLYGAAMRGDRESCLLYVDQLKQRCVEGFKEGSLGIE
                . :. . : ::*  .::.*.**.** .**:**: ::*::* ********: **:*.*.**

SlADC1          QLAAVDAVCDFVSKAIGASDPVRTYHVNLSVFTSIPDFWAIDQLFPIVPIHKLDEHPSAR
SlADC2          QLAAVDSICDWVSKAIGVADPVRTYHVNLSVFTSIPDFWGFSQLFPIVPIHRLDEKPTMR
AtADC1          QLAGVDGLCEWVIKAIGASDPVLTYHVNLSVFTSIPDFWGIDQLFPIVPIHKLDQRPAAR
                ***.**.:*::* ****.:*** ****************.:.*********:**::*: *

SlADC1          GILSDLTCDSDGKIDKFIGGESSLALHELGSG--NSAPYYLGMFLGGAYEEALGGLHNLF
SlADC2          GILSDLTCDSDGKVDKFIGGESSLPLHEIGSG--DGGRYYLGMFLGGAYEEALGGLHNLF
AtADC1          GILSDLTCDSDGKINKFIGGESSLPLHEMDNNGCSGGRYYLGMFLGGAYEEALGGVHNLF
                *************::*********.***:...  ... *****************:****

SlADC1          GGPSVLRVSQSDSPHSFAVTYAVPGPSCADVLRAMQHEPELMFETLKHRAEEFVHKEEE-
SlADC2          GGPSVVRVMQSDSPHSFAVTRSVPGPSCADVLRAMQFEPELMFETLKHRAEEFLEQGEGE
AtADC1          GGPSVVRVLQSDGPHGFAVTRAVMGQSSADVLRAMQHEPELMFQTLKHRAEEPRNNNNKA
                *****:** ***.**.**** :* * *.********.******:********   .: :

SlADC1          ------VEVSLANSLNQSFHNMPYLAPHSSCCFSGYYYCNDENIVGVG-------AECAI
SlADC2          G--EGVAFGSLTSSLAQSFHNMPYLS---SCCFTAEATANANTNTNNGGYYYYSEDNAAA
AtADC1          CGDKGNDKLVVASCLAKSFNNMPYLS----METSTNALTAAVNNLGVYYCDEAAAGGGGK
                          ::..* :**:*****:        :        .  .            .

SlADC1          GEEEFWPYCVA
SlADC2          EEDEIWSY---
AtADC1          GKDENWSYFG-
                 ::* *.*
```

Supplementary Figure 6: Alignment of tomato ADC1 and ADC2 and *A. thaliana* ADC1 peptide sequences, highlighting essential catalytic residues.

Peptide sequences of tomato ADC1 (SlADC1), tomato ADC2 (SlADC2), and *A. thaliana* ADC1 (AtADC1) were aligned using CLUSTALW online tool (https://www.genome.jp/tools-bin/clustalw). Homology between sequences is indicated below alignment, where an asterisk (*) indicates identical residues for all sequences, while dots indicate only partial conservation (. or :). Conserved lysine (K; blue) that binds ADC co-factor, pyridoxal 5-phosphate and cysteine (C; orange) that binds ADC substrate are essential for AtADC1 catalytic activity (Hanfrey et al., 2001) are highlighted, alongside the corresponding SlADC1 and SlADC2 residues.

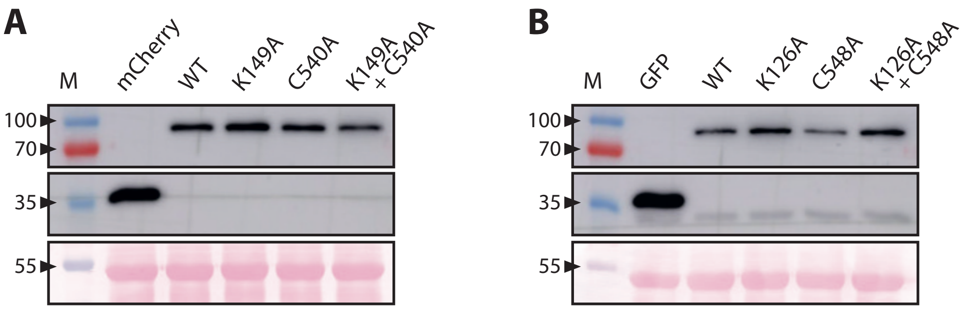

Supplementary Figure 7: All WT and mutant tomato ADC proteins can be transiently expressed in *N. benthamiana* leaves.

A) Immunodetection of transiently expressed SlADC1 WT and mutant variants in N. benthamiana leaves at 2 dpi. HA epitope tagged tomato ADC1 WT, K149A, C540A, and the double mutant K149A + C540A T-DNA constructs were agroinfiltrated into WT *N. benthamiana* leaves alongside the negative control HA-mCherry. Western blot analysis enabled detection of proteins using an anti-HA-horseradish peroxidase (HRP)-conjugated antibody. **B)** Immunodetection of transiently expressed SlADC2 WT and mutant variants in *N. benthamiana* leaves at 2 dpi. Epitope-tagged (myc) tomato ADC2 WT, K126A, C548A, and the double mutant K126A + C548A T-DNA constructs were agroinfiltrated into WT N. benthamiana leaves alongside the negative control, myc-GFP. Detection of proteins using an anti myc primary antibody and conjugated anti mouse HRP secondary antibody. Expected protein sizes are 78 kDa, 80 kDa, and 29 kDa for HA-vSlADC1 variants, myc-SlADC1 variants, and the tagged fluorophores, respectively. Ponceau stained blots show equal protein loading (below each western blot).

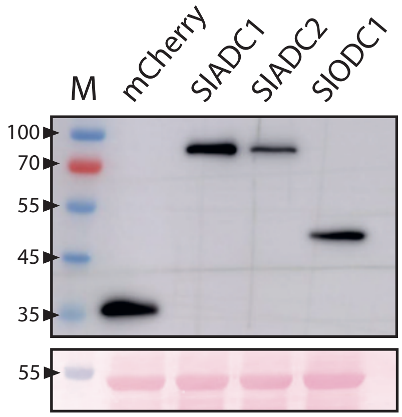

Supplementary Figure 8: Tomato ADC1, ADC2, and ODC1 proteins are expressed in *N. benthamiana* leaves.

Immunodetection of HA epitope-tagged tomato ADC1, ADC2, and ODC1 enzymes and the negative mCherry control transiently expressed in *N. benthamiana* leaves. After 2dpi, samples were harvested for western blot analysis using an anti HA horseradish peroxidase (HRP) conjugated antibody to detect proteins. Expected protein sizes are 78 kDa, 80 kDa, 48 kDa, and 29 kDa for HA-SlADC1, HA-SlADC2, HA-SlODC1, and HA-mCherry, respectively. Ponceau stained blots show equal protein loading (below).

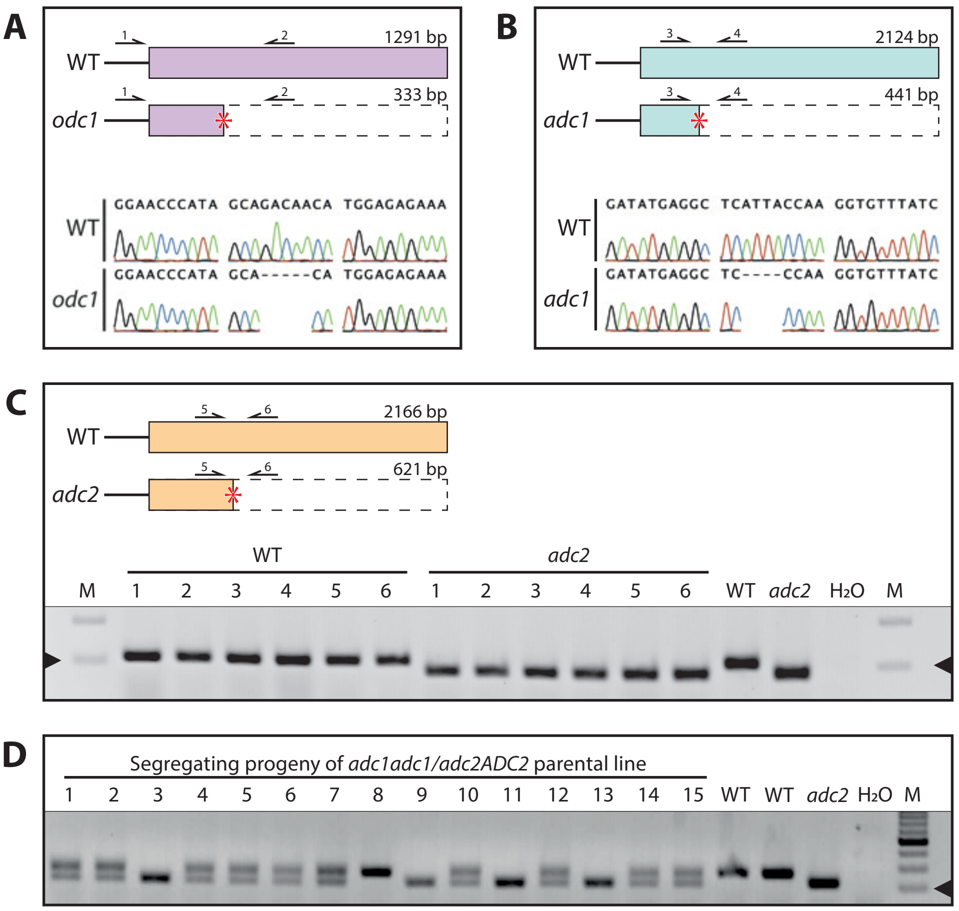

Supplementary Figure 9: CRISPR Cas9 mutagenesis of tomato ODC1, SlADC1, and SlADC2 genes to generate knock out mutants.

A) CRISPR-Cas9 mutagenesis of tomato *odc1* (Sl04g082030.1) resulted in 5 base pair (bp) deletion, causing a frameshift and premature stop codon. Schematic of WT and *odc1* mutant coding sequence (CDS) structure showing premature stop codon (red asterisks) after 333 bp (top). Genotyping was conducted by PCR amplification from extracted gDNA using primers 1 and 2 (black arrows) and sequencing PCR products; trace data from WT and *odc1* mutant shows 5 bp deletion (bottom). **B)** Mutagenesis of *SlADC1* (Sl10g054440.2) resulted in a 4 bp deletion, causing a frameshift and premature stop codon after 441 bp (top). Genotyping was conducted in the same manner with primers 3 and 4; trace data shows 4 bp deletion (bottom). **C)** CRISPR-Cas9 mutagenesis of *SlADC2* (Sl01g110440.4) produced a 52 bp deletion. Schematic of WT and *adc2* mutant CDS structure shows premature stop codon after 621 bp (top). Genotyping was conducted via PCR amplification using primers 5 and 6; amplification of WT produces 272 bp PCR product, while *adc2* produces 220 bp. Previously verified WT and *adc2* genotypes were used as controls, alongside water (H_2O) as a negative PCR control. Black arrow indicates 250 bp band in marker lane. **D)** Generation of *adc1/adc2* double mutant requires genotyping segregating progeny of parental adc1adc1/adc2ADC2 line. Homozygous, Cas9 transgene-free adc1 and adc2 mutants (shown in B and C) were crossed and an *adc1adc1/adc2ADC2* line in the F2 generation was selected. To obtain sufficient *adc1/adc2* plants for phenotyping (i.e., n = 20; Figure 7), 200 progeny plants of this *adc1adc1/adc2ADC2* line were genotyped via PCR amplification of *SlADC2* CDS using primers 5 and 6; typically, only ~10% of progeny pants are adc1/adc2 when using recently harvested seed. Using previously verified WT and *adc2* genotypes as controls, progeny plants homozygous for the *adc2* deletion (i.e., band size 220 bp, verified in two replicate PCR experiments) were selected as *adc1/adc2* plants; for example, plants 3, 9, 11, and 13 here. Black arrow indicates 200 bp band in marker lane.

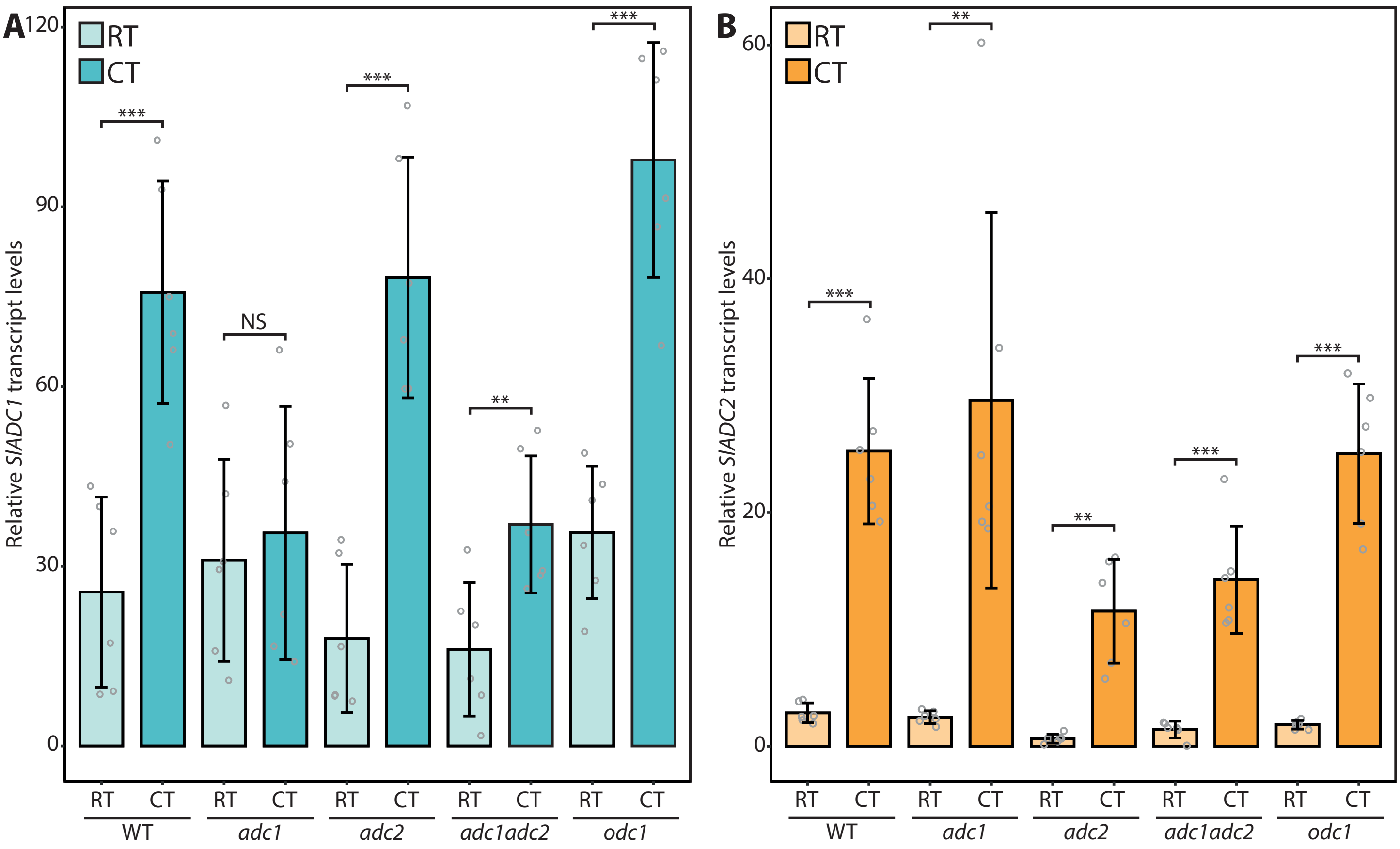

Supplementary Figure 10: Cold treatment of WT, *adc1*, *adc2*, *adc1/adc2*, and *odc1* adult tomato leaves induces SlADC1 and *SlADC2* expression.

RNA extracted from WT *adc1*, *adc2*, *adc1/adc2*, and *odc1* tomato leaves that were cold treated (CT) for 24 hours or the room temperature (RT) controls was used for RT-qPCR to determine **A)** *SlADC1* and **B)** *SlADC2* transcript levels relative to the house keeping SlTIP41 gene expression. Grey circles represent the mean of 3 technical replicates originating from the 6 biological replicates. Statistical significance determined using a Student's T Test to compare expression between each condition from each genotype (a = 0.05); * = P-value < 0.05, ** = P-value < 0.01, and *** = P-value < 0.001.
